# CT-sensitized nanoprobe for effective early diagnosis and treatment of pulmonary fibrosis

**DOI:** 10.1186/s12951-025-03128-0

**Published:** 2025-01-29

**Authors:** Jiwei Hou, Qijian Ji, Tianyu Tang, Yonger Xue, Lin Gao, Li Dai, Jinbing Xie

**Affiliations:** 1 School of Medicine, Nanjing University of Chinese Medicine, Nanjing, 210023 China; 2 Immunology and Reproduction Biology Laboratory & State Key Laboratory of Analytical Chemistry for Life Science, Medical School, Nanjing University, Nanjing, 210093 China; 3 Department of Critical Care Medicine, Xuyi People’s Hospital, 28 Hongwu Road, Xuyi, 211700 Jiangsu China; 4 Department of Emergency Medicine, Jinling Hospital, Medical School of Nanjing University, Nanjing, 210002 PR China; 5 Nurturing Center of Jiangsu Province for State Laboratory of AI Imaging & Interventional Radiology; Basic Medicine Research and Innovation Center of Ministry of Education, Medical School of Southeast University, 87 Dingjiaqiao, Nanjing, 210009 China; 6 Center for BioDelivery Sciences, School of Pharmacy, Shanghai Jiao Tong University, Shanghai, 200240 China; 7 Department of cariol & endodont, Nanjing Stomatological Hospital, Medical School of Nanjing University, Nanjing, 210008 China

**Keywords:** Pulmonary fibrosis, Early diagnosis, Therapeutic window time, Nanoprobes

## Abstract

Early diagnosis is critical for providing a timely window for effective therapy in pulmonary fibrosis (PF); however, achieving this remains a significant challenge. The distinct honeycombing patterns observed in computed tomography (CT) for the primary diagnosis of PF are typically only visible in patients with moderate to severe disease, often leading to missed opportunities for early intervention. In this study, we developed a nanoprobe designed to accumulate at fibroblastic foci and loaded with the CT sensitizer iodide to enable effective early diagnosis of PF. An antibody fragment (Fab′) targeting the platelet-derived growth factor receptor-α, which specifically binds to (myo)fibroblasts, was conjugated to the nanoprobe surface to enhance targeting of fibroblastic foci. Additionally, collagenase was employed to facilitate nanoprobe penetration by degrading the local collagen fibers within these foci. This approach led to significant accumulation of the CT sensitizer iodide in fibrotic lung tissues, resulting in enhanced CT imaging for the detection of fibroblastic foci and enabling early diagnosis of PF. Moreover, a dual-drug combination of oltipraz and rosiglitazone was co-loaded into the nanoparticles for the treatment of early-diagnosed PF. Remarkable therapeutic efficacy was observed in model mice with early PF using these nanoparticles. Our findings present a promising strategy for the early diagnosis of PF, potentially offering a valuable time window for effective treatment of this life-threatening disease.

**Supplementary Information:**

The online version contains supplementary material available at 10.1186/s12951-025-03128-0.

## Introduction

It is widely postulated that the pathogenesis of pulmonary fibrosis (PF) initiates with recurrent injury of the alveolar epithelium. This leads toalveolar inflammation and the subsequent activation of fibroblasts and myofibroblasts, which are collectively referred to as (myo)fibroblasts situated within fibroblastic foci [1, 2]. Activated (myo)fibroblasts are considered the dominant drivers for idiopathic PF (IPF) progression and the main source of excessive extracellular matrix (ECM) protein deposition, particularly collagen [3, 4], which disrupts lung architecture and impairs gas exchange. To the best of our knowledge, no effective clinical diagnosis and corresponding treatment approach for early-stage PF has been reported. While CT is considered the primary diagnostic method for PF in clinic, satisfactory CT signal collection is mostly dependent on pulmonary honeycombing, which only occurs in the moderate to severe-stage PF [5]. Moreover, additional evaluation with surgical lung biopsy is often required to verify the CT diagnostic results, potentially causing significant delays in the accurate and timely theranostics of PF. Patients with moderate to severe-stage PF commonly experience hypoxic injuries to multiple organs, leading to inevitable poor outcomes and increased mortality [6]. Therefore, it is critical to develop an efficient diagnostic strategy for early PF, aiming to generatean essential therapeutic time window in which treatment may yield optimal outcomes. In clinical practice, iodine is often used as a CT contrast agent due to its advantages of exceptional imaging capabilities, minimal dose requirements, and low toxicity [7]. However, the clinical application of iodine-based contrast agents is limited due to significant challenges, such as non-specific distribution and poor penetration into fibrous foci [8]. Thus, delivering iodine into the early fibroblastic foci to enhance the CT signal is expected to facilitate the early diagnosis of PF. Furthermore, the dense fibrotic tissues formed by the deposition and remodeling of ECM proteins, most notably fibrillar collagens, present a substantial challenge for the diffusion and penetration of cell-specific targeting carriers. Therefore, it is highly desirable to promote the penetration and retention of imaging agents and anti-fibrosis drugs into deep fibrotic lung tissues to prompt the accurate theranostics of PF.

Activated (myo)fibroblasts have been proposed to arise from multiple sources during fibrogenesis [9–11]. Numerous studies have demonstrated that the association between oxidative stress and inflammation plays a crucial role in (myo)fibroblast activation [12–14]. It has been reported that excessive reactive oxygen species production promotes persistant activation of (myo)fibroblasts [15, 16]. Nuclear factor-erythroid 2-related factor 2 (Nrf2) is a master regulator of cellular antioxidant and anti-inflammatory responses [17, 18]. Recent studies highlighting the protective role of Nrf2 in fibrotic diseases have suggested that Nrf2 activators may hold great potential in inhibiting fibrogenesis [19, 20]. In addition, lipofibroblasts, the interstitial fibroblasts with resident lipid droplets, have been identified as an important precursor cell of activated (myo)fibroblasts [21]. Of interest, during the fibrosis resolution process, activated (myo)fibroblasts do not undergo apoptotic clearance, but instead transdifferentiate back to lipofibroblasts by initiating a lipogenic program [22]. This reversible transdifferentiation switch between lipogenic and myofibroblastic phenotypes points to a potential direction for stimulating the transition of activated (myo)fibroblasts to lipofibroblasts. Importantly, it has been demonstrated that peroxisome proliferator-activated receptor gamma (PPARγ) activation induced the transdifferentiation of (myo)fibroblast to lipofibroblast [22], indicating that PPARγ agonists might be beneficial in treating IPF.

**Fig. 1:**
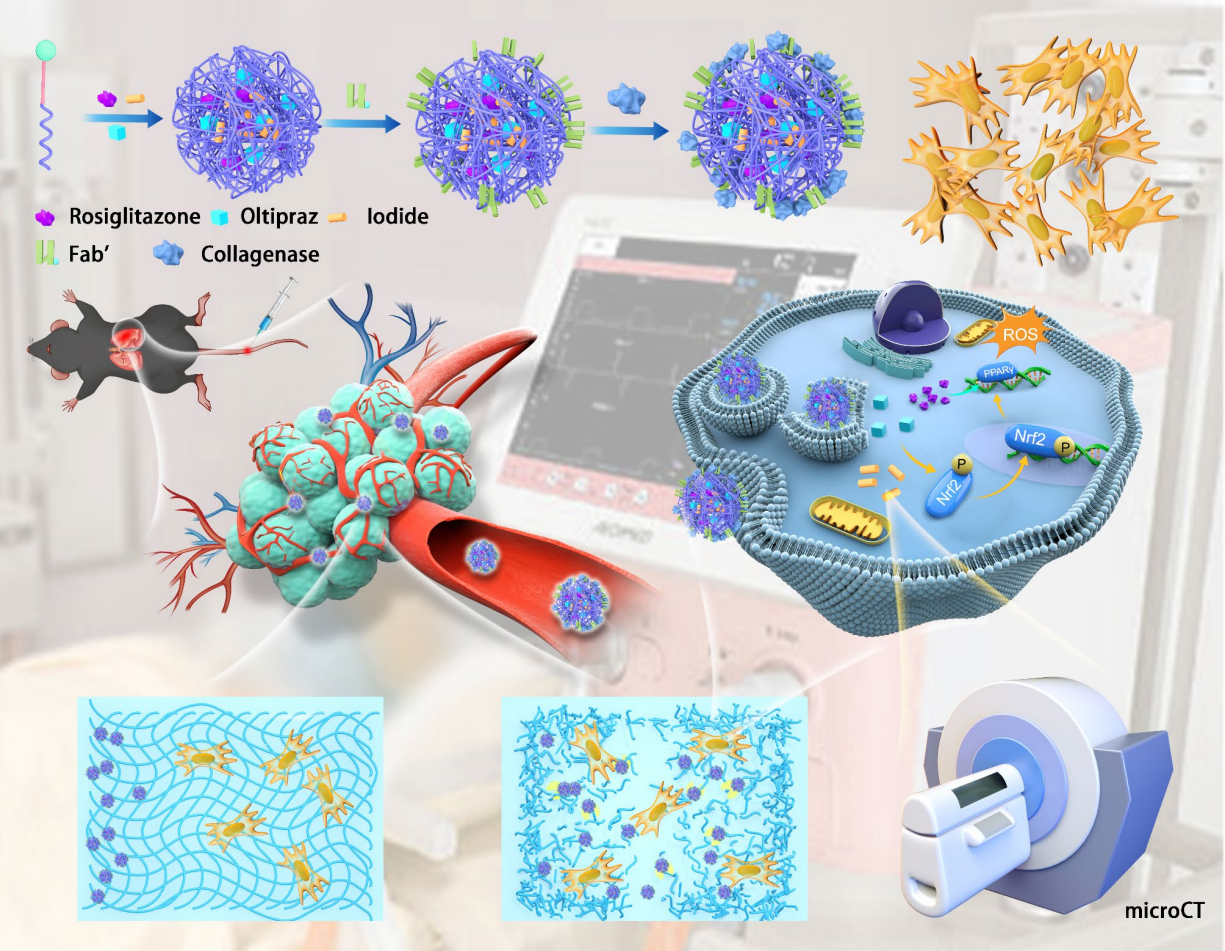
Schematic illustrating the application of fibroblastic foci-accumulated iodide-loaded nanoparticles for the theranostics of early-stage pulmonary fibrosis. (**A**) The synthesis scheme of collagenase-modified iodide-loaded (myo)fibroblast-targeted nanoparticles. Iodide and the dual drugs of oltipraz and rosiglitazone were loaded with polymer MAL-PEG-PAE. The surface of nanoprobes was modified with platelet-derived growth factor receptor-alfa (PDGFRα) antibody fragment (Fab′) for targeting (myo)fibroblast and collagenase for penetration into the fibroblastic foci and promoting the degradation of collagen fibers. The synthesized nanoparticles then simultaneously promote CT imaging measurement and pulmonary fibrosis treatment

As a comprehensive platform, a nanotechnology-based drug delivery system has inherent advantages in the diagnosis of chronic diseases and simultaneous administration of various therapeutic drugs [23–29]. Here, a nanomicelle (collagenase/Fab′@iodide@PEG-PAE nanoprobes, termed CFIPP) loaded with CT enhancer iodide was designed for the early-diagnosis of PF. The Fab′ fragment of the anti-platelet derived growth factor receptor-α (anti-PDGFRα: a (myo)fibroblast-specific receptor) antibody and collagenase, which could degrade the collagen fibers in the fibroblastic foci, was co-conjugated to enhance the penetration and retention of nanoprobes in fibrotic lung tissues. Owing to the high sensitivity of iodide in CT imaging measurement, fibroblast foci and lung fibrosis progression of the PF mouse model were measured using these nanomicelles. Consequently, our finding revealed that the iodide-loaded nanoprobe could facilitate the diagnosis of not only moderate- and severe-stage PF but also early-stage PF. The obtained high contrast signal of this diagnosis strategy was further analyzed by pathological staining and (ex vivo) lung iodine uptake quantification. The accurate diagnosis for early-stage fibroblast foci could provide a potential therapy time window. Thus, dual drugs of oltipraz (an antioxidant drug) and rosiglitazone (a synthetic PPARγ agonist) were encapsulated into the nanomicelle (collagenase/Fab′@iodide@oltipraz/rosiglitazone@PEG-PAE nanoprobes, termed CFIORPP) for the combination therapy of PF. A synergistic therapeutic effect exhibiting significant cure efficiency was observed in the early-stage PF mice treated with the dual anti-fibril drugs-loaded nanomicelles (Fig. 1). Therefore, our work provides not only an early-diagnosis strategy, but also an effective synergistic fibrogenesis inhibition approach for PF.

## Materials and methods

### Materials

Bleomycin was purchased from Nippon Kayaku (Tokyo, Japan). The Nrf2 activators oltipraz and rosiglitazone were purchased from MedChem-Express (no. HY-12519/HY-17386, Monmouth Junction, NJ, USA). Recombinant mouse TGF-β1 was obtained from R&D Systems (Minneapolis, MN, USA; no. 7666-MB-005). LipidTOX red neutral lipid dye was purchased from Invitrogen (no. H34477). N-Hydroxy succinimide (NHS), 1-(3-Dimethylaminopropyl)-3-ethylcarbodiimide hydrochloride (EDC·HCl) were purchased from Shanghai Tansoole technology Co. Ltd., triphenylphosphine were purchased from Biokem Chemical Reagent (Chengdu, China). Collagenase I was obtained from Sigma–Aldrich (St. Louis, MO, USA; no. C5138). Hexane-1,6-dioldiacrylate (HDDA, 99%) and 4,4′-trimethylene dipiperidine (TDP, 97%) were purchased from J&K Scientific Co. Ltd. Antibodies used in this study are listed in Table S1.

### Synthesis of Fab′ from anti-PDGFRα antibody

The Fab′was produced from anti-PDGFRα antibody (no. ab203591, Abcam) according to a reported method [30]. Briefly, about 0.5 mg/mL anti-PDGFRα antibody was digested with pepsin (25 µg/mL, in pH 4.0, 0.1 M acetate buffer) at 37°C, for 8 hours. The solution was purified through ultrafiltration (MWCO = 50 kDa, 10 mM PBS buffer, pH 7.4) to obtain F(ab’)_2_. Then 500 µM DTT was added into the F(ab’)_2_ solution (0.5 mg/mL) with stirring for 0.5 h at 37 °C. The purified Fab′ was collected with a Vivaspin 6 (MWCO = 30 kDa, pH 7.4, 10 mM PBS buffer).

### Synthesis of maleimide-PEG-NH2 (MAL-PEG- NH2)

6-Maleimidocaproic acid (0.126 g, 0.6 mmol), triethylamine (0.182 g, 1.8mmol), EDC (0.346 g, 1.8 mmol) and NHS (0.082 g, 0.72 mmol) were weighted and dissolved in chloroform at ice-bath condition. Then, the solution was added with 0.6 mmol trichloromethane dissolved amino terminated polyethylene glycol segment (NH2-PEG-NH2 ,1.2 g). The mixtrue were stirred for 24 h and then the excess water was added into the solution for 15 min. The organic phase was collected and the organic layer was dried over anhydrous NaSO4, following concentration and recrystallization with ice ethanol. Yield: 1.12 g (about 85% yield).

### Synthesis of maleimide-poly(ethylene glycol)-poly(β-amino ester) (MAL-PEG-PAE)

NH2-PEG-MAL (0.44 g, 0.2 mmol), HDD (0.498 g, 2.2 mmol) and TDP (0.42 g, 2.0 mmol) were dissolved in 20 mL of trichloromethane. After stirred for 72 h at 65 °C under the protection of argon, and the solution was precipitated into excess diethyl ether to obtain MAL-PEG-PAE as measured with a ^1^H NMR spectrometer (JEOL AL 300, Japan) (Figure S1). Yield: 1.22 g (90% yield).

### Synthesis of the iodide-loaded (myo)fibroblast-targeting nanoparticles

For the synthesis of iodide-loaded (myo)fibroblast-targeting nanoparticles, 1 mg iodide, 1.2 mg MAL-PEG-PAE, and 4.8 mg poly(ethylene glycol)-poly(β-amino ester) (PEG-PAE) were mixed and dissolved into 0.4 mL acetone/ethanol solvent (v/v = 1/1). Then 4 mL phosphate buffer solution (PBS, 0.01 mM, pH = 7.4) was droped into the solution at a rate of 1mL/h. The organic solvent in solution was volatiled with stirring under a fume hood to obtain iodide-loaded nanoparticles. Both anti-PDGFRα and collagenase I were conjugated with NHS-CC-SH at molar ratios of Collagenase I/ NHS-CC-SH = 1/5 and anti-PDGFRα/ NHS-CC-SH = 1/5). Then the conjugated anti-PDGFRα and collagenase I was added into the synthesized iodide-loaded nanoparticles solution at molar ratios of Collagenase I/ MAL-PEG-PAE = 1/1 and anti-PDGFRα/ MAL-PEG-PAE = 1/1 to obtain collagenase-modified iodide-loaded (myo)fibroblast-targeted nanoparticles. The non-conjugated Fab′ and collagenase molecules were removed using Spectrum Spectra/Por Biotech Cellulose Ester Dialysis Membrane Tubing (MWCO = 100 kDa, 10 mM of PBS, pH 7.4). The molar ratio of conjugated Fab′ and Collagenase I to nanoparticle was quantified by fluorescence correlation spectroscopy (FCS).

For the cellular uptake experimental, the anti-PDGFRα Fab′ modified with Alexa-488 was used to prepare (myo)fibroblast-targeted nanoparticles. For the in vivo near-infrared imaging experiment, the anti-PDGFRα Fab′ was modified with Alexa-790 to prepare (myo)fibroblast-targeted nanoparticles.

### Dynamic light scattering (DLS) and transmission electron microscopy (TEM) measurement

The size distribution of iodide-loaded (myo)Fibroblast-targeted nanoparticles was measured with a BeNano 180 Zeta Pro (Dandong Bettersize™ Instruments Ltd., Dandong, China) in PBS buffer (10 mM, pH 7.4). About 10 µL of nanoparties solution was droped on a 200-well carbon-coated copper grid to obtain TEM images. The samples on carbon-coated copper grid were then stained with 2.5 wt% uranyl acetate before taking the images with a JEM-1400 (JEOL Ltd., Japan).

### Fluorescence correlation spectroscopy

Fab′-conjugated nanoparticles and Free Cy3-Fab′ was separately dissolved in PBS (pH 7.4, 10 mM, 0.15 M NaCl) with or without 10% serum. Then the FCS measurements were performed with a ConfoCor 3-module equipped CLSM 880 (Carl Zeiss, Germany) and a 40 × water immersion objective. The Cy3 dye ﻿(Nanjing Starleaf Biological Technology Co., China), was excited by argon laser at 514 nm. The diffusion coefficient (D_C_) was calculated from the measured diffusion time normalized to rhodamine 6G (414 µm^2^s^− 1^). Then, the particle size in terms of hydrodynamic diameter (DH) was calculated according to the Einstein-Stokes Eq. (1):

1 $${\rm{DH = }}{{\rm{k}}_{\rm{B}}}{\rm{T/}}{{\rm{3}}_{{\rm{\pi \eta }}}}{{\rm{D}}_{\rm{C}}}$$

T is the temperature, k_B_ is the Boltzmann constant, and η is the viscosity of the solution.

### Collagenase activity

Collagenase activity was measured using a collagenase activity colorimetric assay kit (Aladdin, China). Collagenase, a member of the matrix metalloproteinase family, degrades collagen and facilitates the breakdown of the extracellular matrix. The kit quantifies collagenase activity by utilizing a synthetic peptide (FALGPA), which mimics the structure of collagen. Briefly, 10 µL of free collagenase or collagenase collected from the CFIORPP sample was added to a 96-well plate, followed by 140 µL of collagenase assay buffer and 60 µL of collagenase substrate (FALGPA). The reaction mixture was incubated for 15 min at 37 °C. Collagenase activity was determined by measuring the absorbance at 345 nm using a microplate reader (SpectraMax i3X, Molecular Devices) .

### Cell culture

Isolation and culture of primary normal mouse pulmonary (myo)fibroblasts was performed as previously reported [31, 32]. Freshly isolated fibroblasts were cultured with DMEM/F12 medium (Grand Island, NY, Gibco) containing 15% FBS and 1% penicillin and streptomycin at a concentration higher than 10^5^ cells/mL, and maintained in a humidified atmosphere of 95% air and 5% CO_2_ at 37 °C. The cells were passaged at 1:2 using 0.25% trypsin when they reached 70–90% confluence.

### CCK8 assay

Primary mouse pulmonary fibroblasts were treated with TGF-β1 at 2 ng/mL for 48 h to induce (myo)fibroblastic differentiation. The biological effects of NPs on the viability of (myo)fibroblasts was analyzed by CCK-8 cell counting kit (no. A331-01, Vazyme, Nanjing, China) as described previously [33].

### LC-MS analysis

Mouse pulmonary (myo)fibroblasts were treated with control NPs or dual drugs-loaded NPs for 48 h. The intracellular protein was extracted using lysis buffer containing 4% SDS and 50 mM Triethylammonium bicarbonate (TEABC). Then the protein was digested with trypsin (trypsin: protein = 1:50 (w/w))at 37 °C for 16 h. The peptides were analyzed on Thermo Scientific Q Exactive Plus - Orbitrap mass spectrometer (Thermo Fischer Scientific, Bremen, Germany) connected to Easy-nLC-1200 nano flow liquid chromatography system (Thermo Scientific) as previously reported [34].

### Gene ontology and pathway enrichment analysis

To realize the gene ontology (GO) enrichment analysis, we input the differentially expressed proteins into the Database for Annotation, Visualization and Integrated Discovery (DAVID, https://david.ncifcrf.gov/ (accessed on 2 May 2021). Meanwhile, the functional enrichment analysis tool (FunRich, version: FunRich 3.0, http://www.funrich.org/ (accessed on 2 May 2020) was employed to achieve pathway enrichment analysis. A *p*-value < 0.05 was considered as statistically significant, with the GO results ranked by *p*-value.

### Western blot

Western blot were performed as previously described [35]. The antibodies used were as follows: mouse anti-α-smooth muscle actin (α-SMA), rabbit anti-collagen I, rabbit anti-Nrf2, rabbit anti-PPARγ, rabbit anti-NOX4, and rabbit anti-GAPDH.

### Quantitative real-time polymerase chain reaction (qRT-PCR)

Total RNA extraction was performed as previously described [36]. Primer sequences are shown in Table S2. qRT-PCR was performed using the SYBR Green qPCR Kit (Vazyme no. Q711–02, Nanjing, China) on an ABI ViiA 7 qPCR System (Applied Biosystems, Waltham, MA, USA). The Ct values were analyzed using the 2^−ΔCT^ method, and Porphobilinogen deaminase (PBGD) was used as a reference gene.

### Animals and treatment

Bleomycin-induced mouse IPF model was established as described by us previously [36]. To evaluate the therapeutic effects of the drug-loaded nanoparticles (NPs), mice in the early stage of pulmonary fibrosis were intravenously injected every 3 days with oltipraz-loaded micelles (10 µg oltipraz), rosiglitazone-loaded micelles (10 µg rosiglitazone), or oltipraz/rosiglitazone-loaded micelles (10 µg oltipraz and 10 µg rosiglitazone). Each group consisted of ten mice (*n* = 10). On day 14 following the first nanocarrier instillation, the mice were sacrificed, and lung tissues were collected for further analysis. To analyze the biodistribution of the delivered drugs in vivo, the liver, spleen, heart, kidneys, and lungs were harvested from bleomycin-treated mice 24 h after the first injection of drug-loaded NPs (labeled with FITC, 200 µL, 2.5 mg/kg) (animal live imaging system, ABL X5, Tanon, China).

### Mouse micro-CT image acquisition and analysis

Small-animal micro-CT scans were acquired using an LCT-100 micro‐CT System (Aloka, Tokyo, Japan) 3 h following tail vein injection of Iodine-loaded NPs (Iodine@NPs). The CT density of the lung was quantified by Hounsfield units (HUs) in micro-CT scan analysis. The air density was defined as 1000 and water density as 0. CT images were acquired with the X‐ray source biased at 50 kVp and 1 mA. Mean CT numbers of the maximum cross section in mice lung were calculated with the LCT‐100 micro‐CT System software [37]. The organs were collected after micro-CT scanning, and the iodine content in organs was evaluated through iodine assays. The iodine content in organs was shown as percent injected dose (ID) per gram (% ID/g) of tissue.

### Histology and ashcroft score

Lung sections were subjected to H&E staining (KeyGen no. KGA224, Nanjing, China) for structured observation, or to Masson staining (KeyGen no. KGMST-8003) for the detection of collagen deposits [9]. The degree of pulmonary fibrosis was evaluated by a histopathologists blinded to the experimental groups using the validated semiquantitative Ashcroft method [32, 38]. Briefly, using 100 × magnification, individual fields were assessed by systematically moving over a 32-square grid; each of 10 successive fields was visually graded from 0 (normal lung) to 8 (total fibrous obliteration of the area). The mean value of the grades obtained for all of the areas was taken as the visual fibrotic score.

### Immunofluorescence

Immunofluorescence analyses of cells or lung tissues were performed as described previously [39, 40]. The following primary antibodies were used: mouse anti-α-SMA, rabbit anti-Nrf2, mouse anti-PPARγ, rabbit anti-CD31. Alexa Fluor 488-conjugated goat anti-rat antibody, Alexa Fluor 488-conjugated goat anti-mouse antibody, Alexa Fluor 594-conjugated goat anti-mouse antibody, and Alexa Fluor 594-conjugated goat anti-rabbit antibody (Invitrogen no. A-11006, A-11001, A-11032, and A-11037, respectively, Carlsbad, CA, USA, 1 : 200 dilution) were used as secondary antibodies. Nuclei were stained with 40,6-diamidino-2-phenylindole (DAPI) (Sigma no. D9542).

The images were observed under confocal laser scanning microscope FV3000 (Olympus, Tokyo, Japan).

### Statistical analysis

Data were presented as mean ± SD. Student’s t-test was used for paired comparisons. For the comparison of three or more groups, one-way ANOVA was used for the comparison, which was followed by Duncan’s post hoc test. Statistical significance was defined as *P < 0.05, **P < 0.01.

## Results

### Activity of the PPARγ and Nrf2 pathways increases during lipogenic differentiation of (myo)fibroblasts

**Fig. 2:**
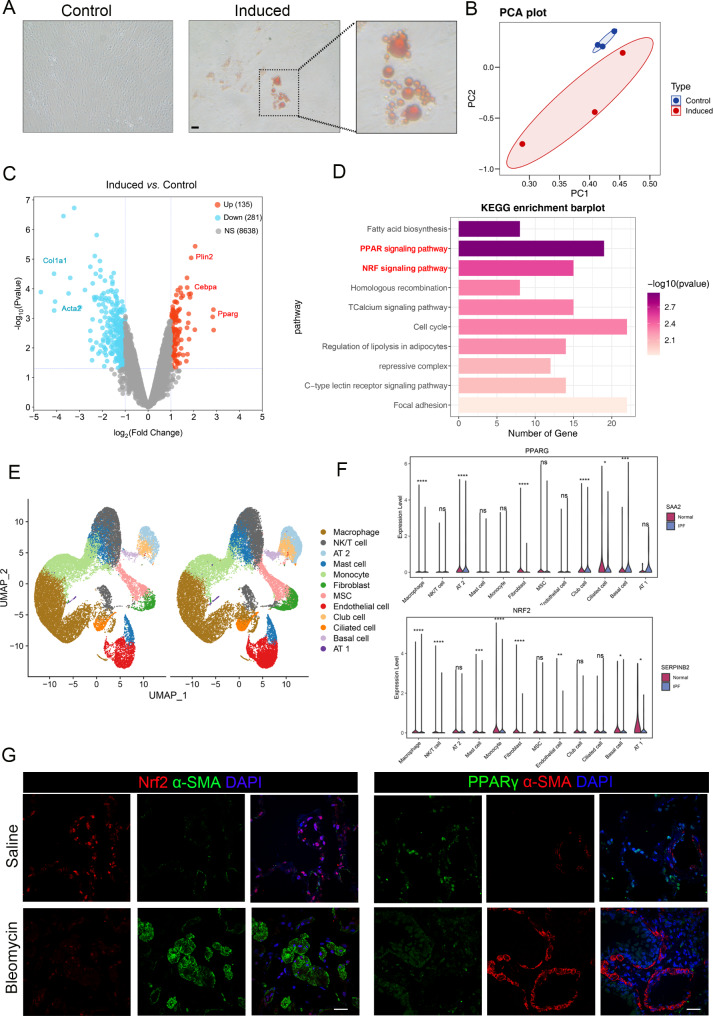
Activity of the PPARγ and Nrf2 pathways increases during lipogenic differentiation of (myo)fibroblasts. (**A**) Mouse lung fibroblasts were cultured in an appropriate differentiation induction medium. After a 21-day lipogenic differentiation period, the cells were stained with oil red O to visualize lipid droplet formation. Scale bar = 20 μm. (**B**) Principal component analysis (PCA) was performed to visualize the distinction between the two groups. (**C**) A volcano plot illustrates the differentially expressed genes (DEGs). (**D**) Bar plots represent the KEGG pathways enriched by the identified DEGs. (**E**-**F**) Integrated scRNA-Seq analysis reveals heterogeneity of normal and fibrotic lung tissues, according to dataset GSE128033. (**E**) Cells on the UMAP plot of all 10 samples were colored as originating from normal and IPF patients. (**F**) Violin plot of PPARG and NRF2 expression in each cell population. *p < 0.05, **p < 0.01, ***p < 0.001, ****p < 0.0001. (**G**) Mice (*n* = 5 per group) received either saline or bleomycin (5 mg/kg body weight) via intratracheal administration. The mice were sacrificed 14 days after bleomycin instillation. The co-localization of α-SMA (a marker for (myo)fibroblasts) with Nrf2 and PPARγ was evaluated through immunofluorescence assays. Scale bar = 50 μm

Inducing (myo)fibroblast lipogenic differentiation is a potential therapeutic strategy for treating IPF. To explore the molecular mechanisms regulating (myo)fibroblast lipogenic differentiation in depth, we established an in vitro induction model for (myo)fibroblast lipogenic differentiation (Fig. 2A). Subsequently, we conducted transcriptome sequencing analysis on cells from both the control group and the induced treatment group. The analysis results showed that after (myo)fibroblast lipogenic differentiation, 135 genes were significantly upregulated, and 281 genes were significantly downregulated compared to the control group (Fig. 2B-C). KEGG pathway enrichment analysis indicated that the differentially expressed genes were primarily enriched in the PPARγ and Nrf2 pathways (Fig. 2D). Consistent with these experimental results, Western blot analysis revealed that the expression of fibrosis marker proteins α-SMA and collagen I was significantly downregulated following (myo)fibroblast lipogenic differentiation (Fig. 2E), while the expression of PPARγ and Nrf2 proteins was also markedly increased (Fig. 2F). Furthermore, we observed a significant decrease in the expression of both PPARγ and Nrf2 proteins in (myo)fibroblasts within fibrotic lung tissues (Fig. 2G, figure S2). Collectively, these results suggested that both Nrf2 and PPARγ have a close correlation with (myo)fibroblast lipogenic differentiation and pulmonary fibrogenesis.

### Preparation and characterization of iodide-loaded (myo)fibroblast-targeted nanomicelles

**Fig. 3:**
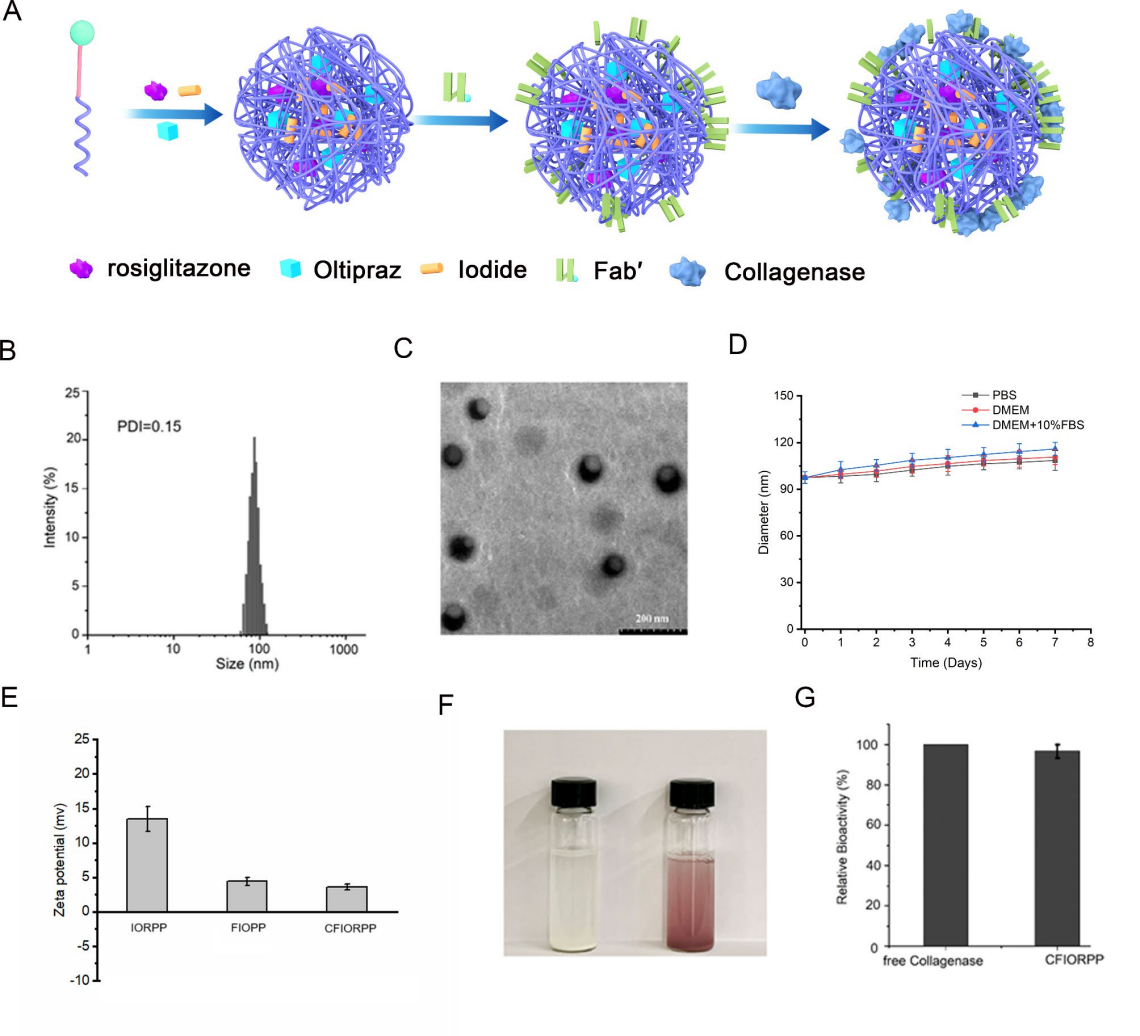
Formation scheme and characterization of collagenase/Fab′@iodide@oltipraz/rosiglitazone@PEG-PAE nanoprobes (CFIORPP). (**A**) CFIORPP was formed *via* the hydrophobic interaction between polymer PAE and iodide, oltipraz and rosiglitazone with conjugation of Fab′ and collagenase. Size distribution and morphology images of CFIORPP resuspended in pH 7.4 PBS as measured with the dynamic light scatterings (DLS) (*n* = 3) (**B**) and TEM (**C**). (**D**) Stability characteristics of CFIORPP in pH 7.4 PBS, DMEM, and DMEM containing 10% FBS for 7 days (*n* = 3). (**E**) Zeta potential of IORPP, FIORPP and CFIORPP that resuspended in pH 7.4 PBS (*n* = 3). (**F**) Photo images of starch solution without (left) or with the addition of CFIORPP. (**G**) The relative bioactivity of free collagenase and the collagenase in CFIORPP

The nanomicelles were formulated by entrapping iodide and dual drugs (oltipraz and rosiglitazone) within a polymer MAL-PEG-PAE through hydrophobic interactions. Then (myo)fibroblast-targeted Fab′ and collagenase were modified on the surface of NPs with a linker of NHS-CC-SH to form CFIORPP (Fig. 3A). The various control NPs, collagenase-modified oltipraz-loaded NPs, collagenase-modified rosiglitazone-loaded NPs, and NPs loaded with collagenase-modified dual drugs (oltipraz and rosiglitazone) were synthesized using an identical procedure. The molar ratio of conjugated collagenase and Fab′ to micelle was 1.75:1 and 1.93:1, respectively, as quantified by FCS. The CFIORPP showed a mean diameter of 97.7 nm as measured by DLS and displayed uniform morphology as visualized by TEM (Fig. 3B-C). The size of CFIORPP has little change in 7 days, which demonstrated that micelle has a high stability (Fig. 3D). The CFIORPP has a slightly positive zeta potential (Fig. 3E). In addition, the starch solution turned blue after CFIORPP was added, indicating that iodine has been successfully loaded into the nanomicelles (Fig. 3F). It should be noted that the collagenase modified on the surface of nanomicelles remained bioactive as measured by the test kit (Fig. 3E). The proteins of Fab’ and the collagenase were analyzed on the matrix assisted laser desorption/ionizationtime-of-flight mass spectrometry (MALDI-TOF-MS) (Figure S3-S4).

**Fig. 4:**
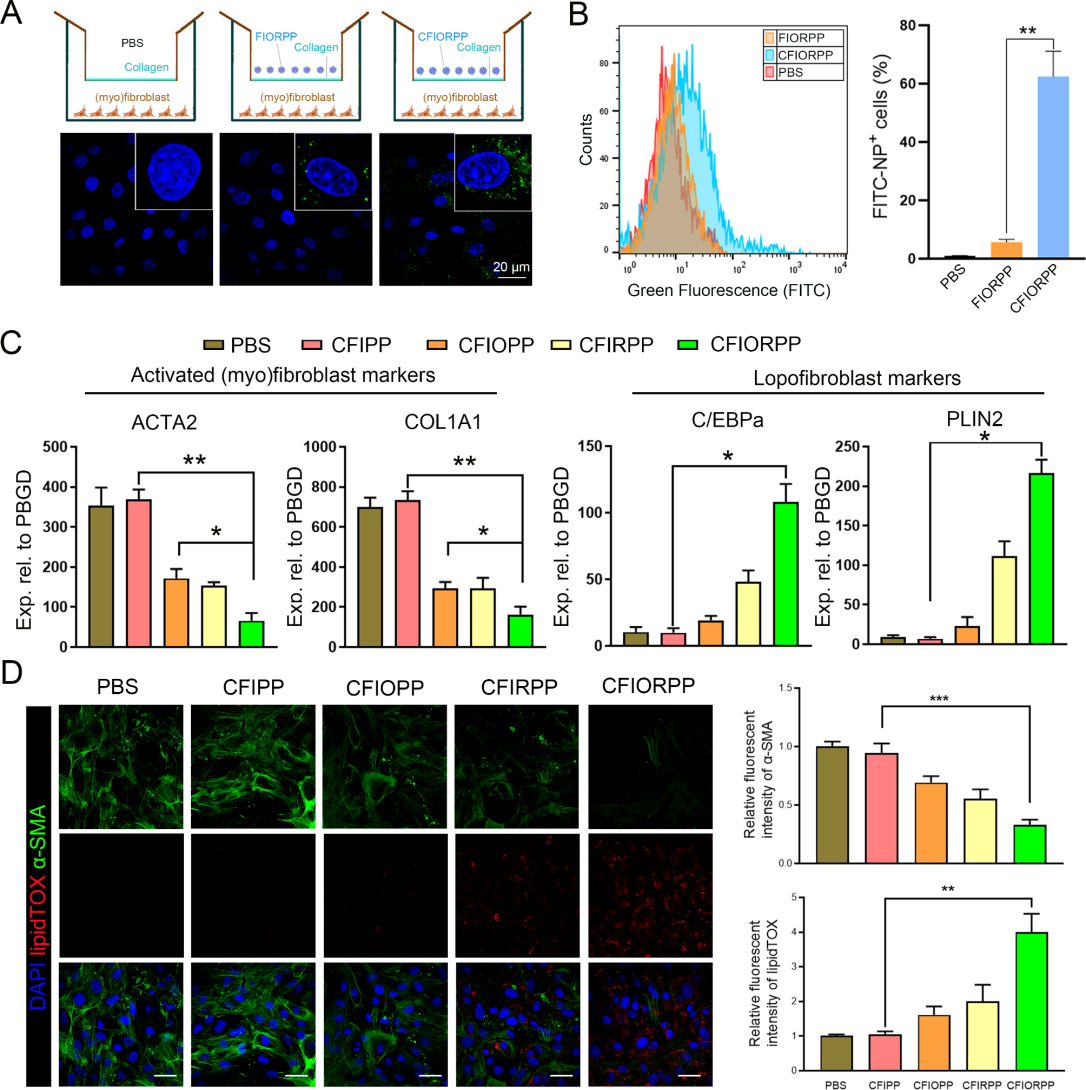
Efficient delivery of dual drugs (oltipraz and rosiglitazone) into (myo)fibroblasts by collagenase/Fab′@iodide@oltipraz/rosiglitazone@PEG-PAE nanoprobes (termed CFIORPP). (**A-B**) A Transwell model was constructed with an upper collagen layer and a lower layer of (myo)fibroblasts, with collagenase-modified nanoparticles (NPs) added to the upper culture medium. (**A**) Intracellular uptake of collagenase-modified NPs in (myo)fibroblasts was determined by immunofluorescence assay. The Fab′ portion of the collagenase-modified NPs was labeled with FITC. (**B**) The percentage of FITC-NP^+^ cells was analyzed by flow cytometry. Right panels show the quantified data for FITC-NP^+^ cells. Results are expressed as the mean ± SD (*p < 0.05). (**C**) The expression of myofibroblast markers (ACTA2 and COL1A) and lipofibroblast differentiation markers (ADRP and PLIN2) in (myo)fibroblasts incubated with various samples was measured by qRT-PCR. Data are expressed as the mean ± SD (**p < 0.01, *p < 0.05). (**D**) Double-staining images of α-SMA and LipidTOX in cells treated with PBS or various NPs. Bar = 50 μm. Right panels show immunofluorescence staining and quantitative statistics of α-SMA and LipidTOX. Data are expressed as the mean ± SD (***p < 0.001, **p < 0.01)

We evaluate the potential cytotoxicity of CFIORPP NPs using (myo)fibroblasts before studying their anti-fibrosis effect. After incubation with (myo)fibroblasts for 24 h, compared with the clinical contrast agent iohexol, iodide-encapsulated micelles showed negligible cytotoxicity (Figure S5). Next, the cellular uptake efficiency of collagenase-modified NPs in (myo)fibroblasts was assessed using a Transwell system. As shown in Fig. 4A and B, (myo)fibroblasts treated with collagenase-modified NPs (CFIORPP) displayed stronger green fluorescence signals compared to those treated with unmodified NPs. This increased fluorescence can be attributed to the collagenase-induced digestion of collagen fibers, which enhanced the cellular uptake of NPs.

### CFIORPP nanoparticles suppress the (myo)fibroblast activity and promote the transdifferentiation of (myo)fibroblast to lipofibroblast

Next, we studied the effect on the expression of Nrf2 and PPARγ in fibroblasts stimulated by various NPs. We incubated the TGF-β1-treated fibroblasts with collagenase-modified oltipraz-loaded NPs (CFOPP), collagenase-modified rosiglitazone-loaded NPs (CFRPP), or NPs loaded with collagenase-modified dual drugs (oltipraz and rosiglitazone) (CFORPP). As expected, the expression of both Nrf2 and PPARγ was asignificantly increased in the cells treated with oltipraz- or rosiglitazone-loaded NPs, as compared to the negative control group (NCPP) (Figure S6). Importantly, it is noteworthy that the cells treated with dual drugs loaded NPs exhibited a simultaneous elevation in the expression of both Nrf2 and PPARγ. The aforementioned results confirmed thesuccessful delivery of dual drugs (oltipraz and rosiglitazone) into fibroblasts using collagenase-modified nanoparticles (NPs), leading to an excellent activation of proteins. Moreover, the expression of activated (myo)fibroblast markers, namely ACTA2 and COL1A1, was significantly downregulated in cells treated with collagenase-modified NPs (Fig. 4C).

The observed data revealed that the (myo)fibroblast differentiation of fibroblast induced by TGF-β1 was also profoundly inhibited by collagenase-modified NPs (Fig. 4C and D). Remarkably, the expression of α-SMA in (myo)fibroblasts treated with NPs loaded with dual drugs (oltipraz and rosiglitazone) was lower than that of oltipraz- or rosiglitazone-loaded NP groups (Fig. 4C). Moreover, the results revealed that the NPs loaded with dual drugs (oltipraz and rosiglitazone) substantially promoted the transdifferentiation of (myo)fibroblast to lipofibroblast, as evidenced by the increased expression of lipogenic markers (C/EBPa and PLIN2) and accumulation of lipid droplets (staining with the neutral lipid dye LipidTOX, Fig. 4C and D).

**Fig. 5:**
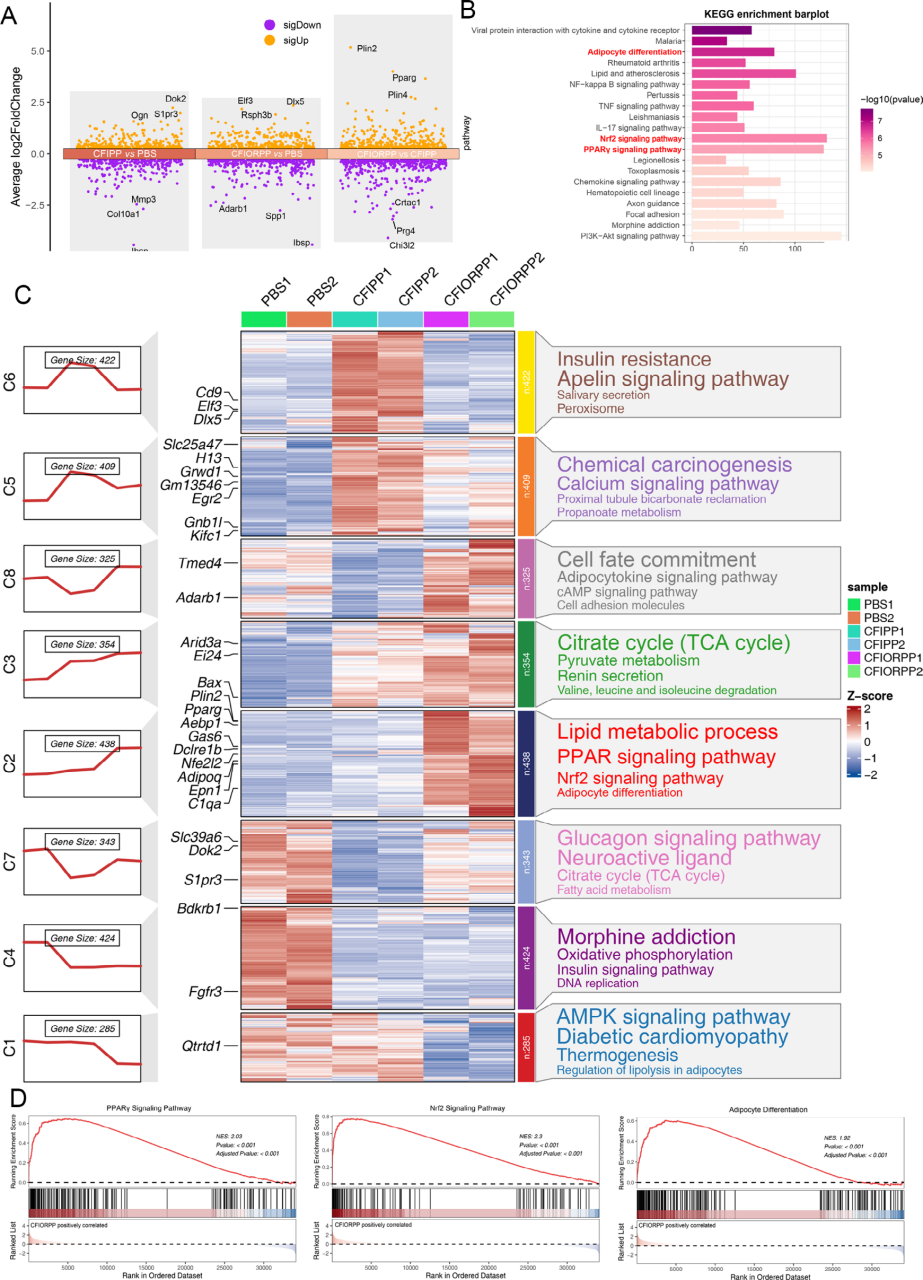
Suppression of fibrotic genes by the collagenase-modified (myo)fibroblast-targeting nanoprobes (CFIORPP) enhances in vitro expression of adipogenic genes. (**A-D**) (Myo)fibroblast treated with collagenase-modified (myo)fibroblast-targeting NPs were subjected to LC-MS analysis. (**A**) A volcano plot showing differentially expressed proteins. (**B**) Kyto Encyclopedia of Gene Ontology (GO) analysis was performed to analyze the enrichment of genes coding DEPs or pathways. (**C**) Left panels: The series of diagrams illustrates the patterns of dynamic changes in representative differentially expressed proteins (DEPs) in each group. Middle panels: heatmap showing representative DEPs between each group. Right panels: representative enriched gene ontology (GO) terms for each group. (**D**) GSEA enrichment plots for representative signaling pathways positively correlated with CFIORPP-treated (myo)fibroblasts

Furthermore, using the liquid chromatography mass spectrometry (LC-MS), we measured the proteome-wide protein expression changes in (myo)fibroblasts treated with collagenase-modified dual drugs-loaded NPs. The results demonstrated that 137 proteins were upregulated and 196 proteins were downregulated in the cells (Fig. 5A). The Gene Ontology (GO), Kyoto Encyclopedia of Genes, and Genomes (KEGG) pathway analysis suggested that the delivery of oltipraz and rosiglitazone to (myo)fibroblasts by collagenase-modified NPs resulted in the overexpression of numerous proteins involved in the Nrf2 and PPARγ signaling pathways as well as tdecreased expression of various proteins closely related to fibrogenic processes (Fig. 5B-C). Consistent with previous reports [41, 42], the KEGG pathway mapping analysis indicated that Nrf2 and signaling pathway might be associated with the enhanced myogenic-to-lipogenic switch in fibrogenic phenotype (Fig. 4B). Consistently, gene set enrichment analysis (GSEA) also suggested that CFIORPP-treated (myo)fibroblasts expressed high levels of genes involved in the the Nrf2 and PPARγ signaling pathways, and adipocyte differentiation (Fig. 5C-D). The proteome-wide results demonstrated that the activation of Nrf2 and PPARγ could simultaneously suppress the myofibroblast activation and promote the transdifferentiation of (myo)fibroblast to lipofibroblast. This mechanism suggests a promising strategy for the mitigation of PF.

### CFIORPP efficiently penetrated in fibrotic lung tissues and targeted to (myo)fibroblasts

**Fig. 6:**
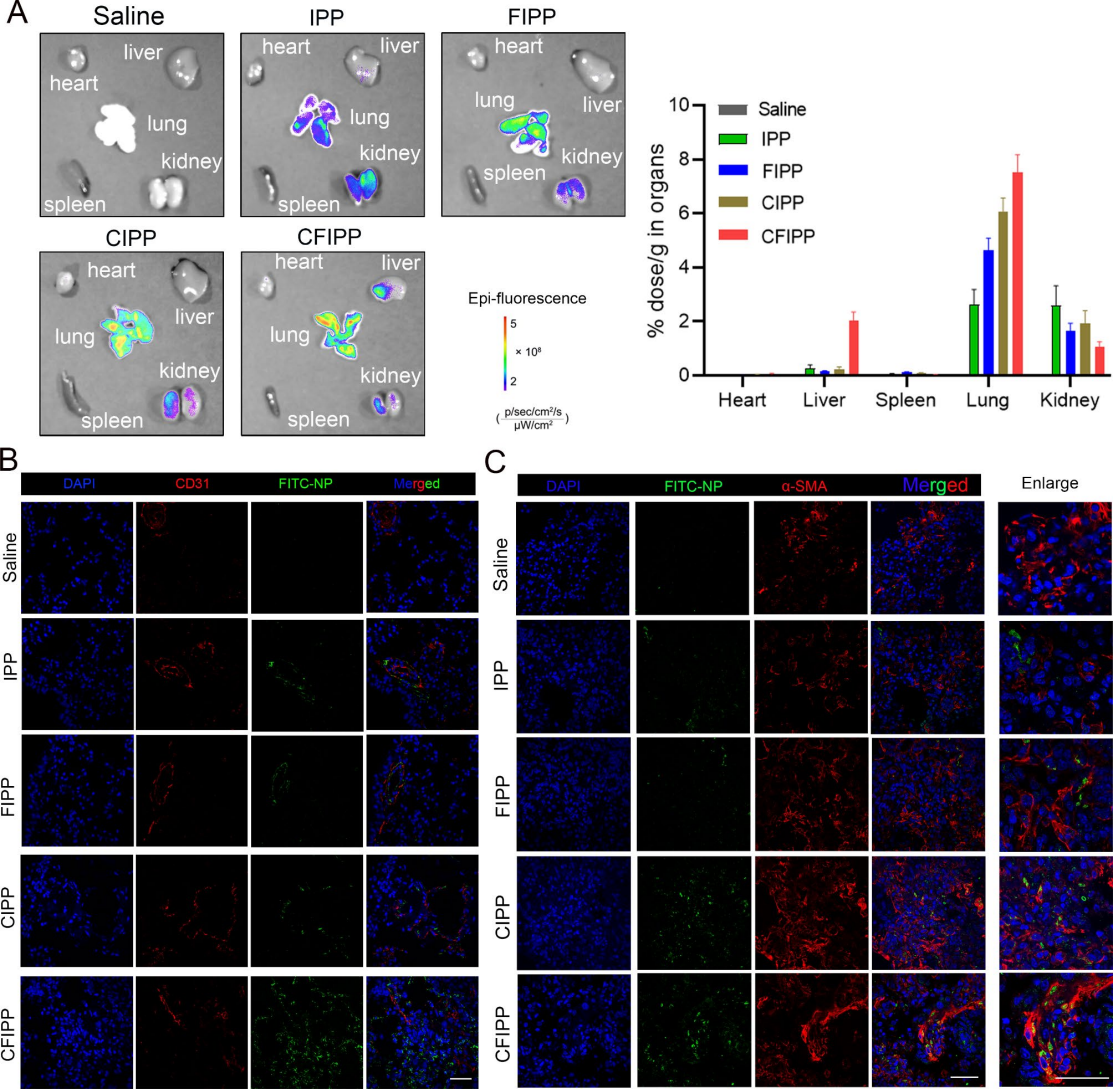
Biodistribution of collagenase-modified nanoparticles (NPs) after intravenous injection. (**A**) Ex vivo NIR fluorescence images of major organs (lungs, heart, liver, spleen, and kidneys) of bleomycin-induced PF mice were obtained at 24 h post-injection of NPs with or without collagenase modification. (**B**) Immunofluorescence staining images of the collagenase-modified NPs distribution in fibrotic lung tissues. The FITC-labeled NPs are shown in green, vascular endothelial cells are labeled with the CD31 antibody and shown in red, and the cell nuclei are stained with DAPI (blue). Bar = 50 μm. (**C**) The colocalization of FITC-NP^+^α-SMA^+^ (myo)fibroblasts in the lung tissues of negative control-NPs (NC-NPs) or collagenase-modified NPs treated mice was showed by immunofluorescence assay. Bar = 50 μm. Right panels: quantified data of FITC-NP^+^ (myo)fibroblasts. Results are expressed as the mean ± SD (*n* = 5; *p < 0.05)

Here, the biodistribution of delivered NPs in the lungs was evaluated. The nanoprobes with (CFIORPP) or without collagenase modification (FIORPP) were separately intravenously injected into the bleomycin-induced fibrotic mice. As shown in Fig. 6A, the accumulation of CFIORPP in lung tissue is significantly greater compared to FIORPP. This finding provides compelling evidence that the conjugation of collagenase to the nanoprobes greatly enhances their ability to penetrate fibrotic tissue. In addition, fibrotic lung tissues from mice receiving these treatments were further observed by confocal microscopy. Notebly, in the extravascular regions over 100 μm away from vessels, the red fluorescence signals were distributed more widely in the CFIORPP-treated mice than FIORPP group (Fig. 6B), sugggesting that the collagenase-modified NPs obtain superior penetration efficiency in fibrotic lung tissues by degrading collagen. We further investigated the detailed distribution and localization of the NPs inside the lung tissue. The immunofluorescence analysis provided compelling evidence that the collagenase-modified NPs delivered more drugs into the α-SMA^+^ (myo)fibroblasts compared to the NPs without anti-PDGFRα Fab′ modification (Fig. 6C). These results further indicated that dual modification of collagenase and anti-PDGFRα Fab′ greatly improved the penetration, distribution, and (myo)fibroblast targeting of NPs in fibrotic lung tissue.

### Iodide-encapsulated (myo)fibroblast-targeting NPs facilitate the early diagnosis of PF model mouse

(Myo)fibroblast foci is a hallmark of PF and suitable to be the target for PF diagnosis [43]. However, the small area of fibrotic lesions accumulated in the early stage of PF can not be easily detected under CT examination, posing significant challenges for the early diagnosis of PF. In this regard, we hypothesized that the iodide-loaded (myo)fibroblast-targeting NPs could be advantageous for the early diagnosis of PF by amplifying the CT signal coming from fibrotic lesions. Therefore, we first investigated whether iodine@NPs monitor disease progression in the bleomycin-induced mouse model of PF. Following intratracheal spray of bleomycin, the disease progressed in a stepwise fashion, as determined by H&E and Masson’s trichrome staining (Figure S7A). Hydroxyproline, the main component of collagen and some proteins, is a crucial index of collagen deposition in lung tissues [44]. It was found that collagen deposition (as determined by the hydroxyproline content) and histological Ashcroft scoring of lung fibrosis increased progressively over time (Figure S7B and C). To assess the reliability of iodine content as a representative indicator of disease progression, we exmained the iodine content in the mice treated with bleomycin on days 7 and 14. As shown in Figure S7D and E, iodine accumulation in the lungs of mice augmented as the disease progressed. Additionally, a significant positive correlation was observed between iodine accumulation and the mean CT number in the lungs (Figure S7F).

**Fig. 7:**
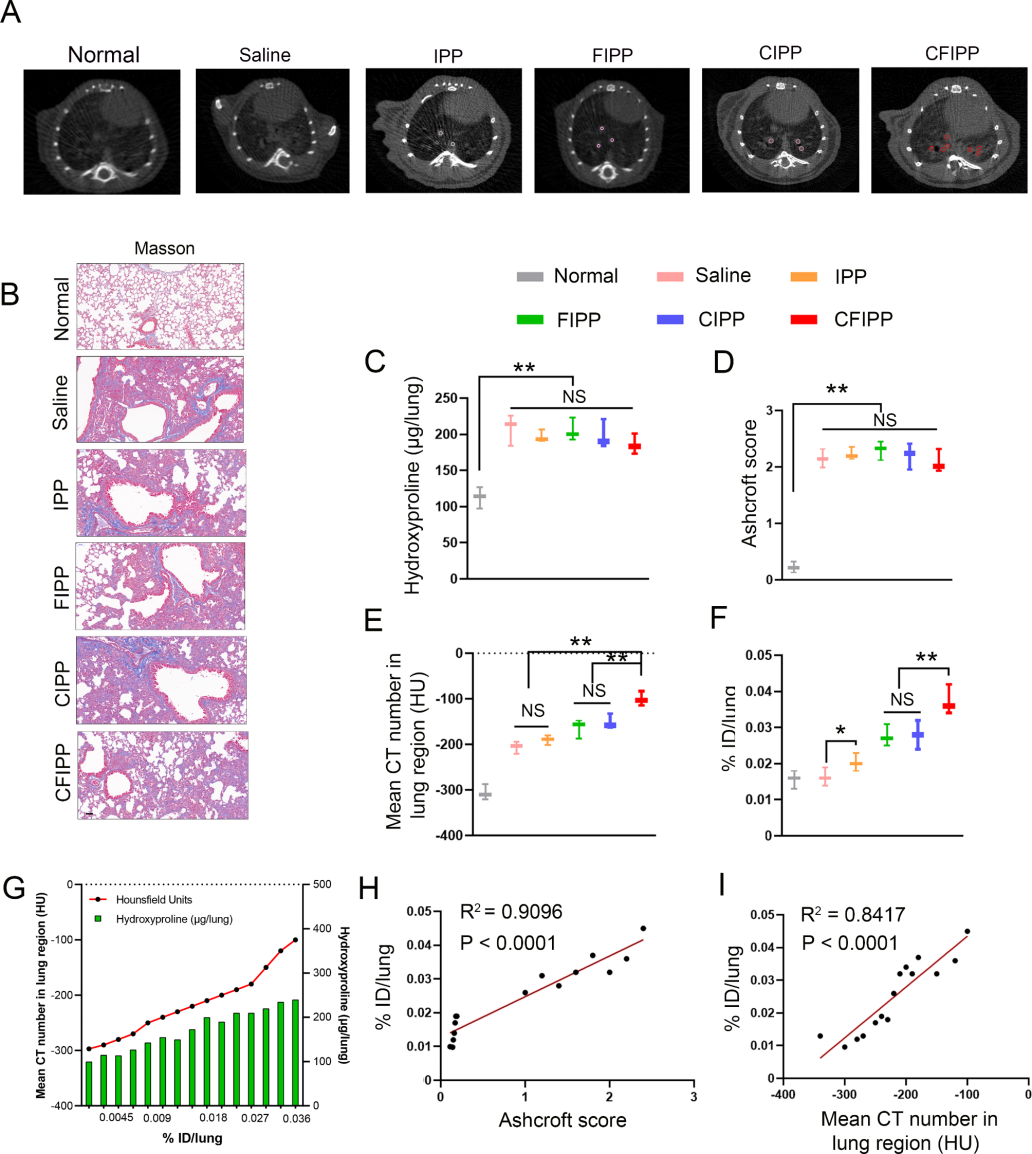
Collagenase/Fab′@iodide@PEG-PAE nanoprobes (termed CFIPP) detect stages of disease in a mouse model of pulmonary fibrosis. (**A-I**) Representative micro-CT (**A**) and Masson’s trichrome staining images (**B**) of the lungs of bleomycin-treated mice administrated with the IPP and CFIPP. Micro-CT analysis was performed 3 h after iodine@NP injection. Circles represent areas with high CT values. Bar = 20 μm. (**C**) The content of hydroxyproline (HYP) was evaluated through HYP assay. Results are expressed as the mean ± SD (*n* = 5; *p < 0.05). (**D**) The quantification of pulmonary fibrosis was shown as an Ashcroft score (*n* = 5; mean ± SD; **p < 0.01). (**E**) The quantification of the mean CT numbers of the lung region. Results are expressed as the mean ± SD (*n* = 5; *p < 0.05). NS, no significant. (**F**) Uptake of iodine in lungs from IPP- and CFIPP-treated mice 120 min after injection expressed as % ID/lung. Data are expressed as the mean ± SD (*n* = 5; *p < 0.05). NS, not significant. (**G**) The trend chart of the mean CT number in the lung region and hydroxyproline content with changes in iodine uptake in the lung. (**H-I**) Correlation between ex vivo iodine uptake in the lung and the Ashcroft score (**H**) as well as the mean CT number in the lung region (**I**)

The biodistribution of delivered collagenase/Fab′@iodide@PEG-PAE nanoprobes (CFIPP) in the lungs was measured to evaluate the CT image-enhancing effect of CFIPP. Neither hydroxyproline content nor Ashcroft scoring showed a significant difference among different groups of bleomycin-treated mice, regardless of whether they received administration of CFIPP or negative control-NPs (NC-NPs) (Fig. 7A-D). The mean CT number of CFIPP-treated mouse lung tissues was significantly enhanced, compared with that of the lung tissues of the groups treated with NC-NPs (Fig. 7E). This index was therefore used to define the severity of fibrosis in mice and distinguish between early (− 150 to − 100), middle (− 100 to − 50), and advanced stages (more than − 50) of PF. It was found that CFIPP could accumulate specifically in fibrotic lungs of bleomycin-treated mice but not in the healthy mice (Fig. 7F). Moreover, the iodide content in lung tissues was positively correlated with collagen deposition, Ashcroft scoring of lung fibrosis, and the mean CT number of lung tissues (Fig. 7G − I). These results indicated that iodide-encapsulated (myo)fibroblast-targeting nanocarriers effectively enhanced the CT signal of fibrosis mice, thus holding great promise for PF theranostics.

### The iodide-encapsulated, dual drugs-loaded (myo)fibroblast-targeting nanocarriers accelerated the resolution of lung fibrosis in early PF mice

Encouraged by the promising in vitro results of collagenase-modified dual drugs loaded NPs suppressing the (myo)fibroblast activity and promoting the transdifferentiation of (myo)fibroblast to lipofibroblast, we next investigated whether delayed administration of collagenase-modified NPs could alleviate the progression of bleomycin-induced PF. It is widely recognized that severe fibrosis symptoms typically occur 10 days after administration of bleomycinand progressively worsen over time. Thus, 10 days after bleomycin injectoin, we intravenously injected Collagenase/Fab′@iodide@oltipraz/rosiglitazone@PEG-PAE nanoprobes (CFIORPP) every 3 days. However, delayed administration of CFIORPP did not achieve the anticipated therapeutic effects. As measured using Masson’s trichrome staining, administration of CFIORPP on the 10th day after bleomycin treatment has no obvious suppression effect on the collagen deposition in alveolar regions and airways of mice (Figure S8A and B). Additionally, the corresponding survival of PF mice was not notably prolonged (Figure S8C). The mice treated with CFIORPP showed a slightly higher survival rate (38.4%) than those treated with saline (18.3%) or FIORPP (19.1%). Theseresults indicated that confident early diagnosis of PF could provide a new critical time window for effective treatment.

**Fig. 8:**
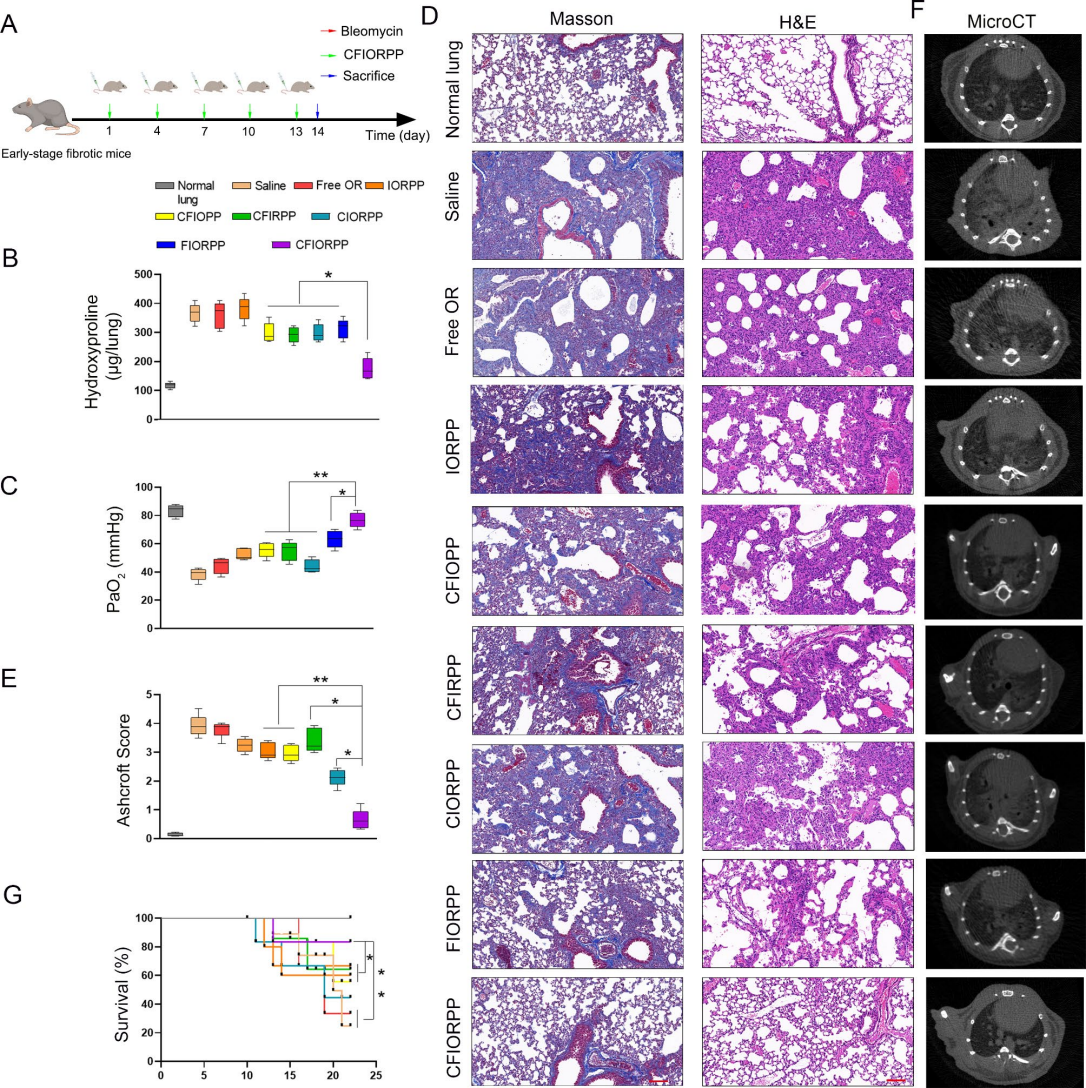
The therapy effects of collagenase-modified (myo)fibroblast-targeting nanoparticles (NPs) on bleomycin-induced pulmonary fibrosis. Mice in the early stage of pulmonary fibrosis were intravenously injected with Collagenase/Fab′@iodide@oltipraz/rosiglitazone@PEG-PAE nanoprobes (termed CFIORPP) every 3 days. (**A**) Schematic diagram of the collagenase-modified (myo)fibroblast-targeting NPs therapy procedure. (**B**) The content of hydroxyproline (HYP) was evaluated through HYP assays. Data are expressed as the mean ± SD (*n* = 5; *p < 0.05; **p < 0.01). (**C**) Arterial partial pressure (PaO_2_) was assessed by blood gas analysis. Values are expressed as the mean ± SD (*n* = 5; *p < 0.05; **p < 0.01). (**D**) Pulmonary fibrosis was determined by H&E staining, and collagen I was visualized by Masson trichrome staining. Bar = 100 μm. (**E**) The progress of pulmonary fibrosis was represented by the quantification of the Ashcroft score. Values are expressed as the mean ± SD (*n* = 5; *p < 0.05; **p < 0.01). (**F**) Representative images of micro-computed tomography (micro-CT) axial sections of lungs of mice on the 21st day after bleomycin instillation. (**G**) Kaplan–Meier analysis curve represents the survival of normal mice (control) or mice treated with saline or single drug loaded or dual drug loaded NPs. Values are expressed as the mean ± SD (*n* = 10; *p < 0.05, **p < 0.01)

As such, we reasonably proposed that administration of the dual drug loaded NPs at an early stage of PF may achieve optimal therapeutic effects. To this end, we first screened the mice in the early stage of fibrosis by using the mean CT number (− 150 to − 100). Next, we intravenously injected CFIORPP every 3 days (Fig. 8A). As expected, mice treated with CFIORPP showed significantly reduced collagen deposition (Fig. 8B). In addition, CFIORPP treatment caused much higher arterial partial pressure (PaO_2_) in the lung tissues of bleomycin-induced PF mice, compared to the saline-treated group and the groups treated with NPs loaded with a single drug (oltipraz or rosiglitazone) (CFIRPP/CFIOPP), suggesting the satisfactory recovery of lung function in CFIORPP-treated mice (Fig. 8C). Moreover, the lung function of mice treated with collagenase-modified dual drug loaded NPs has an enhanced recovery from fibrosis as evidenced with the H&E as well as Masson’s trichrome staining (Fig. 8D). Correspondingly, histopathology and the quantification of lung fibrosis showed a significant decrease in the extent of fibrosis from 3.1% ± 0.12% and 2.9% ± 0.23% in the CFIRPP- and CFIOPP-treated groups, respectively, to 0.73% ± 0.08% in the CFIORPP-treated group (Fig. 8E). Consistently, the results of micro-CT measurement also demonstrated that lung fibrosis was greatly attenuated in the mice treated with CFIORPP (Fig. 8F). Furthermore, collagenase-modified dual drug loaded NP-treated mice exhibited a much higher survival rate (81.4%) than those mice treated with the single drug-loaded NP, either CFIOPP (58.6%) or CFIRPP (63.4%) (Fig. 8G). Collectively, these results suggested that early diagnosis with iodide-loaded (myo)fibroblast-targeted NPs provides a key time window for effective therapy of PF, and that the dual activation of Nrf2 and PPARγ signaling accelerated fibrosis resolution and had a synergistic therapeutic effect on bleomycin-induced PF.

To evaluate the biosafety of these collagenase-modified NPs, we examined the major organs (heart, heart, liver, spleen, and lung) after the therapy experiment. The H&E staining results showed no apparent histopathological lesions in organ samples of the collagenase-modified NP-treated group compared with the saline group (Figure S9). These results indicated the superior biocompatibility of the collagenase-modified dual drug-loaded NPs.

## Discussion

Despite recent advances in our molecular understanding of the pathogenesis of PF, there is still a significant lack of effective approaches for early diagnosis and treatment. The activation of collagen-secreting (myo)fibroblasts, the primary fibrogenic cells, plays a critical role in exacerbating the progression of fibrosis [45, 46]. The irreversible lung damage caused by disease progression emphasizes the importance of early treatment. Consistently, a late PF diagnosis is associated with a worse prognosis. accurate early-stage diagnosis and the identification of novel therapeutic targets are essential strategies for optimizing clinical management. However, PF presents with general respiratory symptoms, which may cause a significant number of patients to be initially misdiagnosed with chronic obstructive pulmonary disease, asthma, or pneumonia, among other diseases [47, 48]. Diagnosis of PF requires the presence of a pattern of usual interstitial pneumonia (UIP) on high-resolution CT (HRCT), and the limitations of this approach include moderate interobserver agreement on HRCT [5]. The irreversible lung damage caused by disease progression underscores the importance of early treatment. An early diagnosis of PF is of increasing importance, and a window of opportunity may exist during which treatment can have optimal therapeutic effects. Imaging of PF is limited to CT which is often not sufficient for a definite diagnosis. Many patients with suspected PF present with atypical high-resolution CT characteristics but are unfit for surgical lung biopsy, therefore preventing a confident diagnosis [49]. The state of the art suggests an iterative, multidisciplinary process that incorporates available clinical, laboratory, imaging, and histological features [47]. A potential strategy that may promote the early diagnostic efficacy of imaging agents entails the use of a colloidal NP system [50, 51].

NPs are emerging drug-delivery systems with a high potential for effective andaccurate targeting ability of diseased tissues. Among various biomaterials for constructing drug-loaded nanocarriers, cationic polymer-based nanomaterials have shown promising advantages owing to their unique physicochemical properties [52, 53]. However, dense fibrotic tissues with notable fibrillar collagens caused by the deposition and remodeling of ECM proteins pose a challenge to the diffusion of these drug delivery nanocarriers. Here, we developed a collagenase-modified polymer micelle to enhance the retention of nanomedicine in deep fibrotic lung tissue. The modified collagenase promotes the digestion of collagen fibers like a scavenger, which enhances the penetration and accumulation of NPs in fibrotic lung tissues. Future research can focus on how to avoid the possible denaturing of the collagenase protein during the modification process and to maintain a therapeutic release rate at the target site. As such, we loaded iodide, a molecule that can enhance the CT signal, onto these nanoprobes. When the iodide-loaded NPs (iodide@NPs) was used to the early-PF mice, we observed a strong correlation of lung uptake of iodide with hydroxyproline content, indicating the ability of iodide@NPs to detect early-stage PF and to monitor disease progression. However, it is worth noting that the higher baseline abundance of collagens and fibroblasts in healthy lungs and other target tissues may result in higher background. Overall, in this study, we first screened mice in the early stage of PF by using iodide@NPs, which successfully identified the early diagnosis of PF, thus providing a possible time window for effective PF treatment. In our study, we recognized the potential impact of beam hardening and streaking artifacts on the accuracy of CT-based nanoprobe imaging. Previous research has proposed various methods for artifact correction, including image filtering techniques, deep learning models, and statistical optimization strategies [54–56]. Specifically, the proposed segmentation-free empirical beam hardening correction (sfEBHC) method addresses beam hardening and streaking artifacts by deforming the histogram of the reconstructed CT image, enabling correction without the need for tissue segmentation, and effectively improving image quality even in the presence of high noise and strong artifacts [57]. Additionally, certain methods combine algorithms iteratively to further enhance image quality [58]. In future research, we plan to adopt these methods, particularly those combining deep learning and image processing, to address artifact correction more effectively and improve image quality in our studies.

The role of PF pathogenesis has been widely recognized [59–61]; however, the mechanisms by which oxidative stress contributes to the formation of fibroblast foci remain elusive. Nrf2 has been widely investigated as a key transcription factor that regulates cellular antioxidant and anti-inflammatory responses. Considering that Nrf2 plays an essential role in host protection against oxidative injury [62], we propose that this transcription factor regulates the profibrotic phenotype of (myo)fibroblasts. Here, the aberrant reduction of Nrf2 during the (myo)fibroblastic activation of fibroblast and the overexpression of Nrf2 in fibroblast that attenuated TGF-β1-mediated (myo)fibroblastic activation suggested that Nrf2 could be a valuable therapeutic target of PF. It was reported that oltipraz (4-methyl-5-(pyrazinyl-2) − 1-2-disulfide-3-thione) is an effective Nrf2 agonist, owing to its strong antioxidant activity, and it is easy to accumulate in organs due to its lipophilicity [63–65]. Recent studies have shown that olitipraz has a therapeutic effect on liver cirrhosis, fatty liver, and tumorigenesis, which makes it a good prospect in clinical application [66, 67]. Of note, the (myo)fibroblastic activation of fibroblast is a complex crosstalking biological process in which several pathways might be involved, and single protein-targeted therapeutic effects are limited. The PPARγ agonist rosiglitazone can greatly counteract the TGF-β1-mediated myogenic differentiation in primary human lung fibroblasts [22, 68]. We speculated that simultaneously promoting the expression of Nrf2 and PPARγ by delivering dual drugs (oltipraz and rosiglitazone) to fibroblasts might present a better therapeutic effect. This was proven by the significantly lower levels of collagen deposition and fibroblastic foci within lung tissues in the mice with early stage PF treated with the (myo)fibroblast-targeted dual drugs (oltipraz and rosiglitazone) loaded NPs. These results of remarkable therapeutic effect indicated that Nrf2 and PPARγ may play cooperative roles in the pathogenesis of PF. Note that the treatment performed at the advanced stage of PF cannot hinder the progression of the disease, indicating the importance of the earlier diagnosis of PF.

An interesting and noteworthy finding from our study is the potential regulatory relationship between Nrf2 and PPARγ. Previous studies have demonstrated that Nrf2 deficiency leads to a reduction in PPARγ expression in hepatocytes, suggesting a possible interaction between these two key molecules [69]. Consistent with these findings, our study reveals that the Nrf2 agonist Oltipraz can enhance the expression of PPARγ, further supporting the notion of a functional relationship between Nrf2 and PPARγ. This interaction may have important implications for understanding the molecular mechanisms underlying diseases such as IPF. Given the potential role of Nrf2 in regulating PPARγ expression, future research exploring the molecular mechanisms of this interplay could provide valuable insights and uncover new therapeutic targets for IPF intervention.

Different drug administration routes, including oral, nebulized, and transdermal delivery, can profoundly influence the diagnostic and therapeutic outcomes of nanomedicines [70]. Nebuliser systems, in particular, is highly suitable for the treatment of pulmonary diseases such as PF, as it facilitates direct drug deposition in the lungs, enabling non-invasive, targeted therapy that enhances therapeutic efficacy while minimizing systemic side effects [71, 72]. However, challenges remain in achieving effective aerosolization and optimizing lung deposition of nebulized nanomedicines [73]. Future advancements in particle size, surface properties, and solvent systems may help address these issues, positioning nebulized nanomedicines as a promising therapeutic strategy for pulmonary disorders. In the present study, however, we opted for intravenous injection to ensure a precise evaluation of the in vivo distribution and therapeutic efficacy of the nanomedicines. Further investigation into nebulized drug delivery approaches may be warranted in subsequent research. Inaddition, although we have conducted in vivo assessments, including biochemical and histopathological analysis, showing a favorable biosafety profile, the evaluation of long-term effects and potential toxicity of repeated nanoprobe administration is still limited. In future studies, it will be important to extend the duration of nanoprobe administration and include additional evaluation criteria, such as detailed organ toxicity assessments, inflammatory responses, and monitoring of chronic effects. These comprehensive evaluations will be crucial to better understand the safety profile of repeated nanoprobe use and to ensure their safe and effective application in clinical settings.

## Conclusion

In conclusion, we developed a multifunctional NPs system for PF diagnosis and therapeutics. The surface modification of collagenase and the Fab′ fragment of (myo)fibroblast-targeted anti-PDGFRα antibody greatly enhanced the accumulation in fibroblastic foci. Importantly, the iodide encapsulated within NPs effectively enhanced the CT signal thereby contributing to the early diagnosis of PF, which provides a key time window for disease treatment. The NPs loaded with dual drugs (oltipraz and rosiglitazone) achieved a remarkable therapeutic effect on bleomycin-induced PF via dual activation of Nrf2 and PPARγ signaling. Our work paves the way for early-stage PF theranostics.

## Electronic supplementary material

Below is the link to the electronic supplementary material.

Supplementary Material 1

## Data Availability

No datasets were generated or analysed during the current study.
