# Supplementary material for: CT-sensitized nanoprobe for effective early diagnosis and treatment of pulmonary fibrosis
Source: J Nanobiotechnology. 2025 Jan 29;23:60. doi: 10.1186/s12951-025-03128-0 (PMC11776250; doi:10.1186/s12951-025-03128-0)
Supplement: Supplementary file 1 — Supplementary Material 1 [file 12951_2025_3128_MOESM1_ESM.docx]

**CT-sensitized nanoprobe for effective early diagnosis and treatment of pulmonary fibrosis**

Jiwei Hou^a,b#^, Qijian Ji^c,d, #*^ , Tianyu Tang^e#^, Yonger Xue^f*^, Lin Gao^e^, Li Dai^g^, Jinbing Xie^e*^

^a^ School of Medicine, Nanjing University of Chinese Medicine, Nanjing, 210023, China.

^b^ Immunology and Reproduction Biology Laboratory & State Key Laboratory of Analytical Chemistry for Life Science, Medical School, Nanjing University, Nanjing, 210093, China.

^c^ Department of Critical Care Medicine, Xuyi People's Hospital, 28 Hongwu Road, Xuyi, 211700, Jiangsu, China.

^d^ Department of Emergency Medicine, Jinling Hospital, Medical School of Nanjing University, Nanjing, 210002, PR China.

^e^ Jiangsu Key Laboratory of Molecular Imaging and Functional Imaging, Medical School of Southeast University, 87 Dingjiaqiao, Nanjing 210009, China.

^f^ Center for BioDelivery Sciences, School of Pharmacy, Shanghai Jiao Tong University, Shanghai, 200240, China.

^g^ Department of cariol & endodont, Nanjing Stomatological Hospital, Medical School of Nanjing University, Nanjing 210008, China.

^#^These authors contributed equally to this work.

***Corresponding authors:** Qijian Ji (jiqj_nj@sina.com)

Yonger Xue ([Xue.462@osu.edu](mailto:Xue.462@osu.edu))

Jinbing Xie ([xiejb@seu.edu.cn](mailto:xiejb@seu.edu.cn))

**Table S1.** Specifications of primary antibodies

Vendor Antibody Catalog no. Working dilution

Cell Signaling Rabbit anti-PDGF Receptor α #3174 WB, 1:1000 IF, 1:500

Rabbit anti-PPARγ #2435 WB, 1:1000 IF, 1:500

Rabbit anti-Nrf2 #12721 WB, 1:1000 IF, 1:500

Abcam Rabbit Anti-α-SMA ab124964 IF, 1:50

Rabbit Anti-CD31 ab222783 IF, 1:50

Boster Rabbit anti-Collagen I BA0325 WB, 1:1000

Mouse anti-α-SMA BM0002 WB, 1:1000 IF, 1:50

Mouse anti-GAPDH BM3876 WB, 1:5000

**Table S2.** Primers used for real-time PCR.

Primer sequence (5’-3’)

Accession Target

Number gene Formard Reverse

NM_007392.3 ACTA2 CAGCCATCTTTCATTGGGATGGA TGGTACCCCCTGACAGGAC

NM_007742.4 Col1a1 ATGTTCAGCTTTGTGGACCTC CTGTACGCAGGTGATTGGTG

NM_010902.4 Nrf2 GGTTGCCCACATTCCCAAAC TATCCAGGGCAAGCGACTCA

NM_001127330.2 PPARγ TTGCTGTCATTATTCTCAGTGGA GAGGACTCAGGGTGGTTCAG

NM_007408.3 PLIN2 TCAGCTCCATTCTACTGTTCACC CCTGAATTTTCTGATTGGCAC

NM_001287514.1 C/EBPa AATGGCAGTGTGCACGTCTA CCCCAGCCGTTAGTGAAGAG

NM_001110251.1 PBGD TGTCTGGTAACGGCAATGCG CCCACGCGAATCACTCTCAT


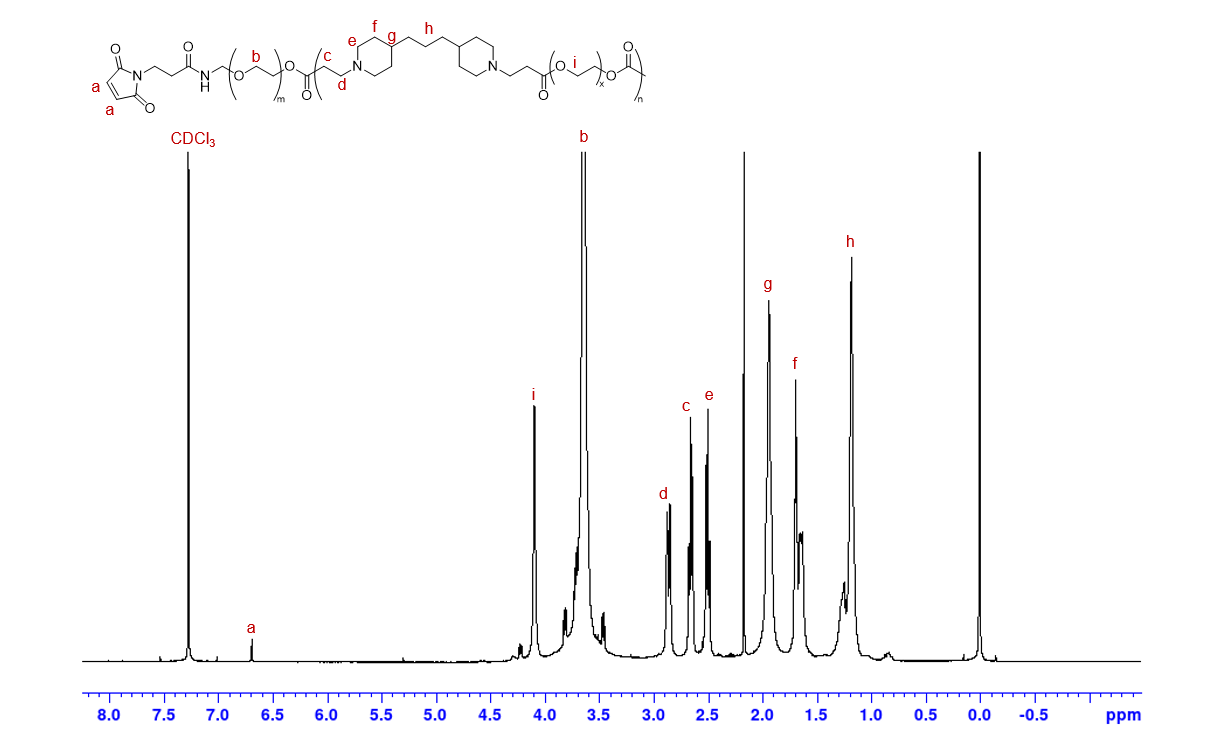


**Supplementary Figure S1.** **The ^1^H NMR spectrum of Mal-PEG-PAE.**


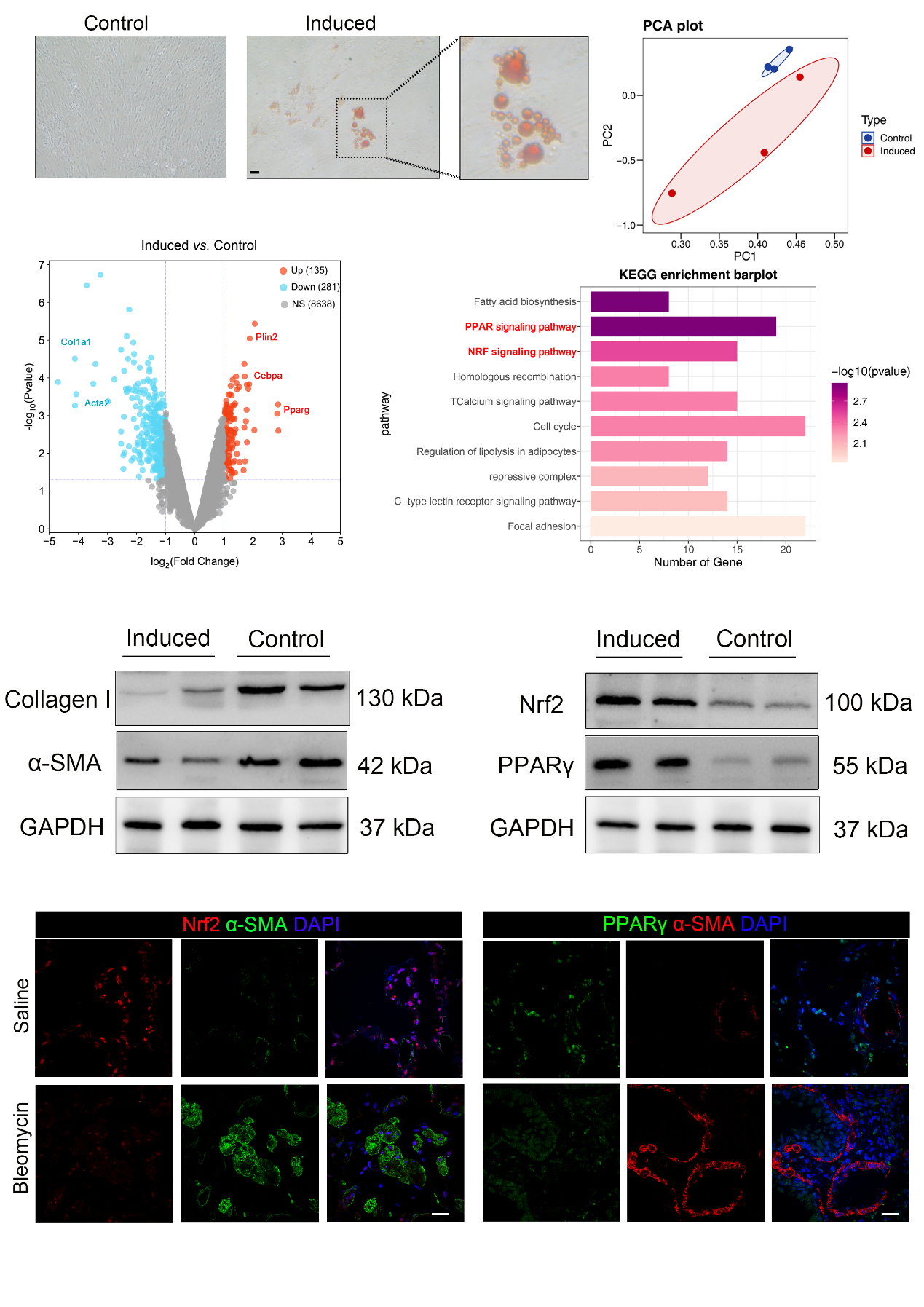


**Supplementary Figure S2.** Mouse lung fibroblasts were cultured in an appropriate differentiation induction medium. After a 21-day lipogenic differentiation period, the expression levels of α-SMA, Collagen I, Nrf2, and PPARγ were assessed using Western blot analysis.


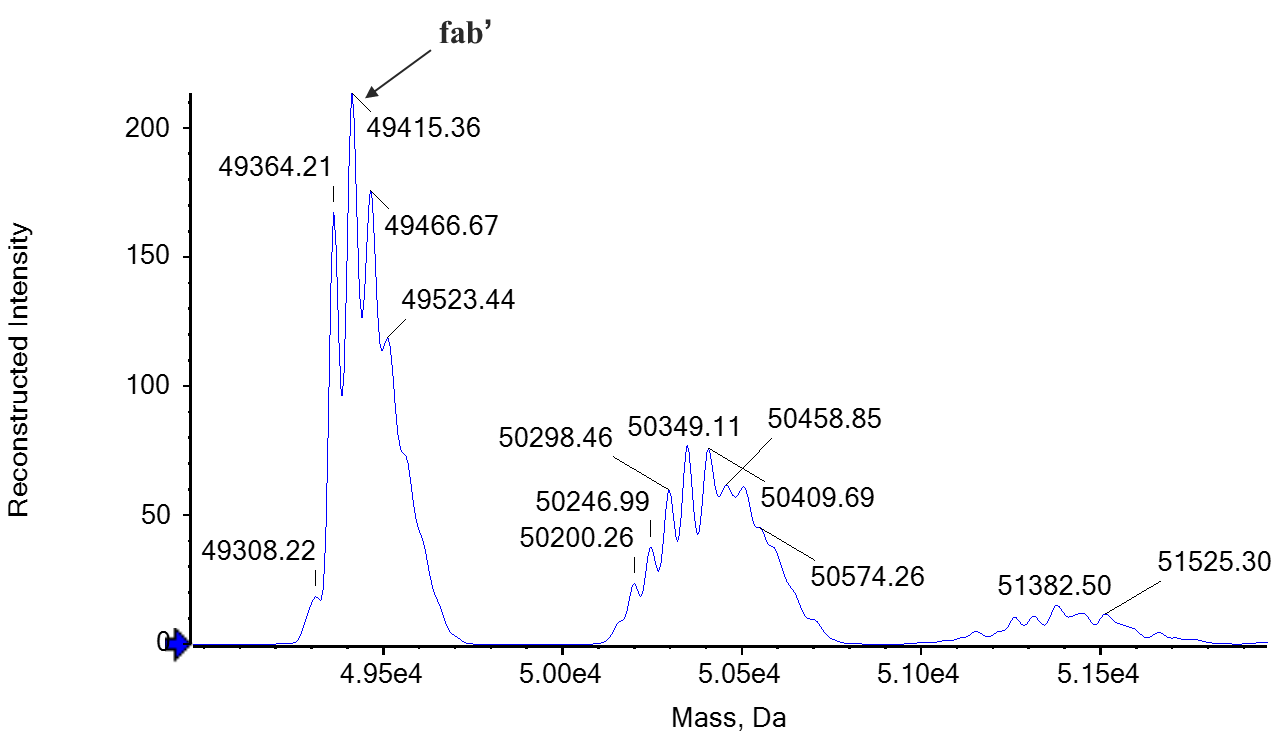


**Supplementary Figure S3. The mass spectrum of Fab'.**


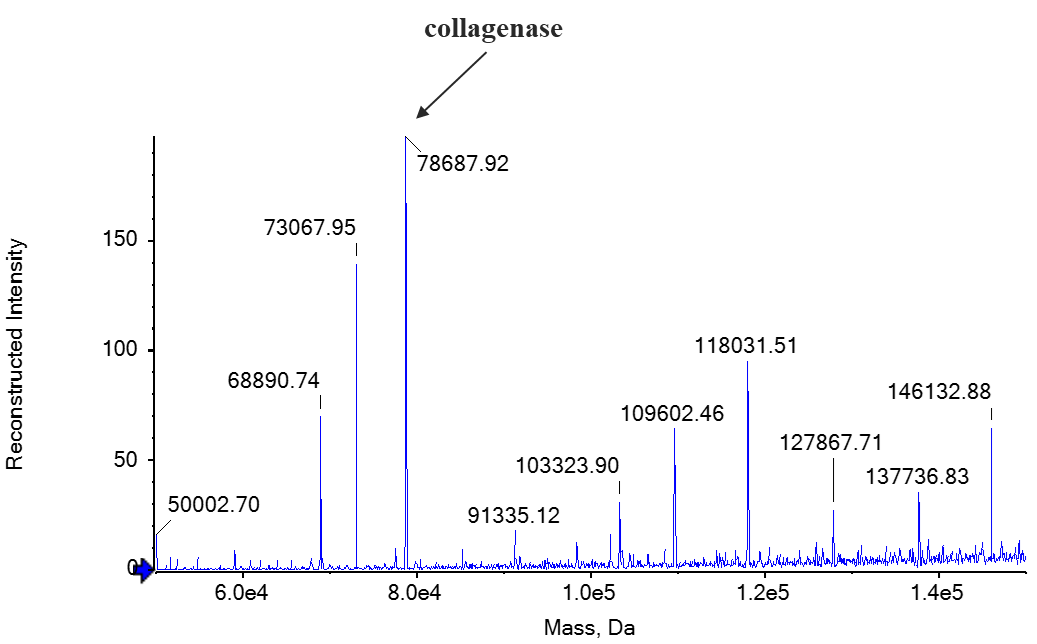


**Supplementary Figure S4. The mass spectrum of collagenase.**


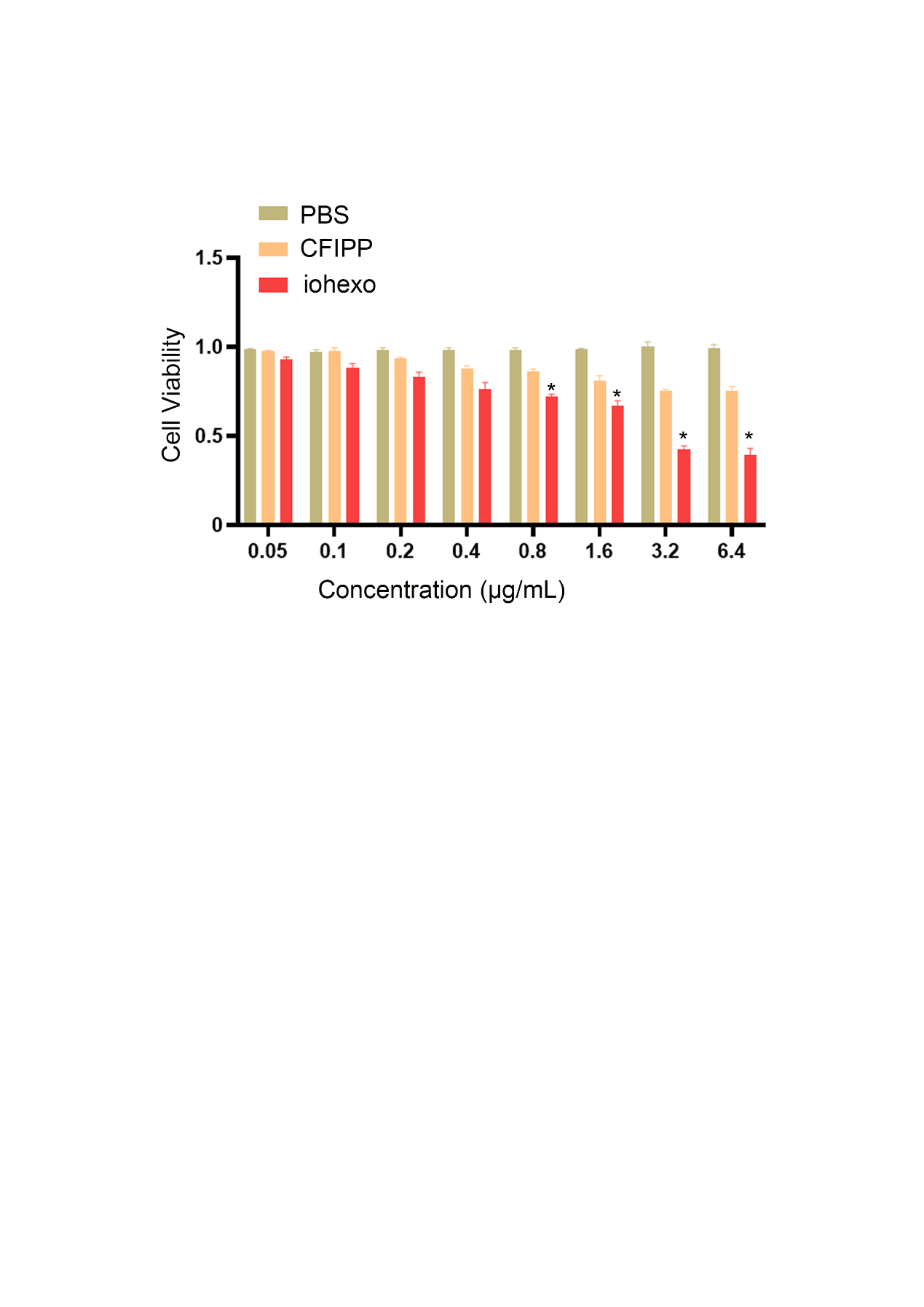


**Supplementary Figure S5. Cytotoxicity of the Collagenase/Fab’@iodide @PEG-PAE nanoprobes (termed CFIPP)**. The cells viability of (myo)fibroblasts incubated with various concentrations of PBS, CFIPP, and iohexo for 24 h as measured by CCK-8 assay. The data are presented as the means ± SD (*p < 0.05, compared with collagense-NPs group).


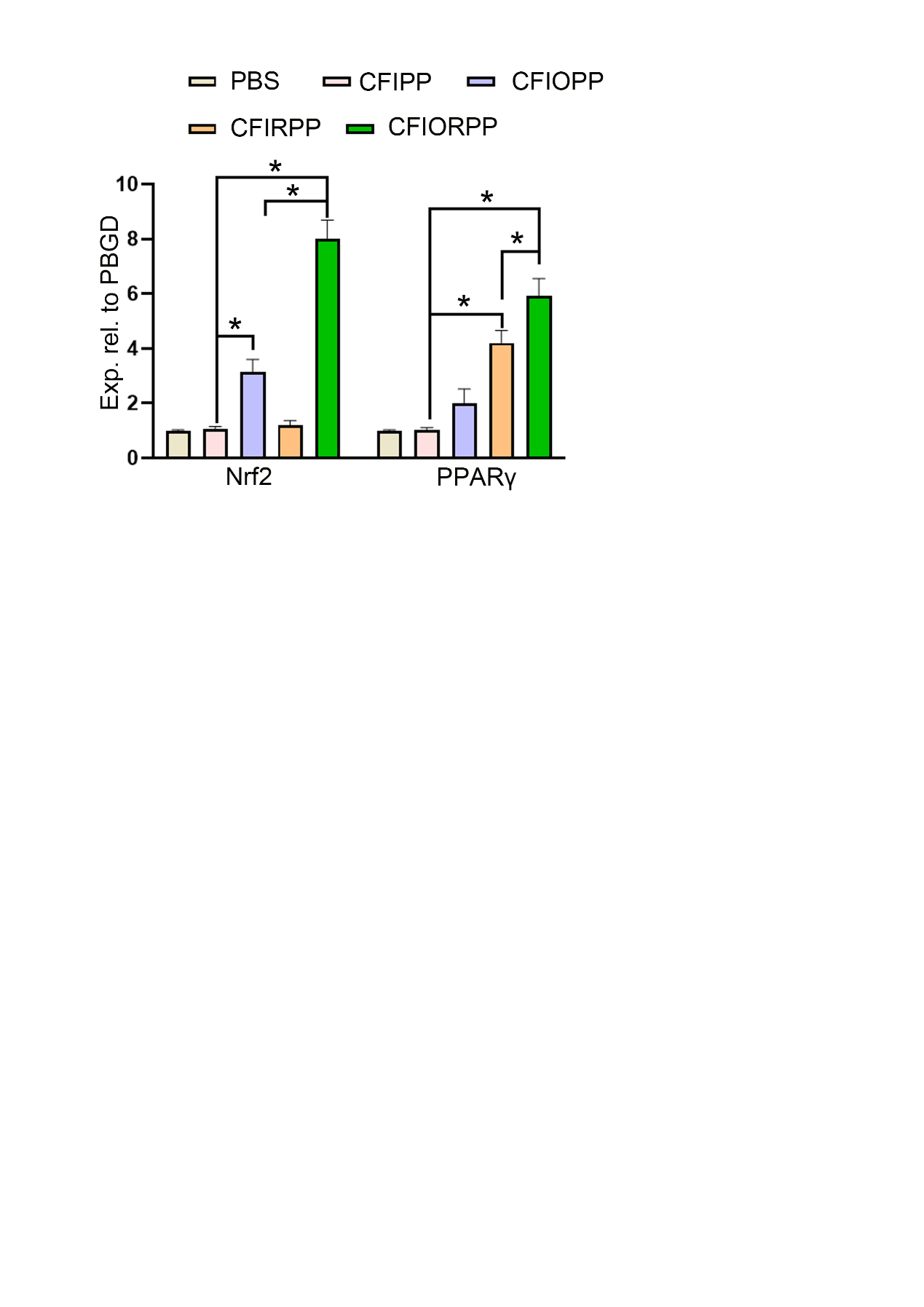


**Supplementary Figure S6. Codelivery of dual drugs by Collagenase/Fab’@iodide@oltipraz/rosiglitazone@PEG-PAE nanoprobes (termed CFIORPP) promotes Nrf2 and PPARγ expression in *in vitro* (myo)fibroblasts.** Nrf2 and PPARγ mRNA levels in (myo)fibroblasts treated with collagenase-modified loaded with different drugs (oltipraz or rosiglitazone) were measured by qRT-PCR. Data are shown as the mean ± SD (*p < 0.05).


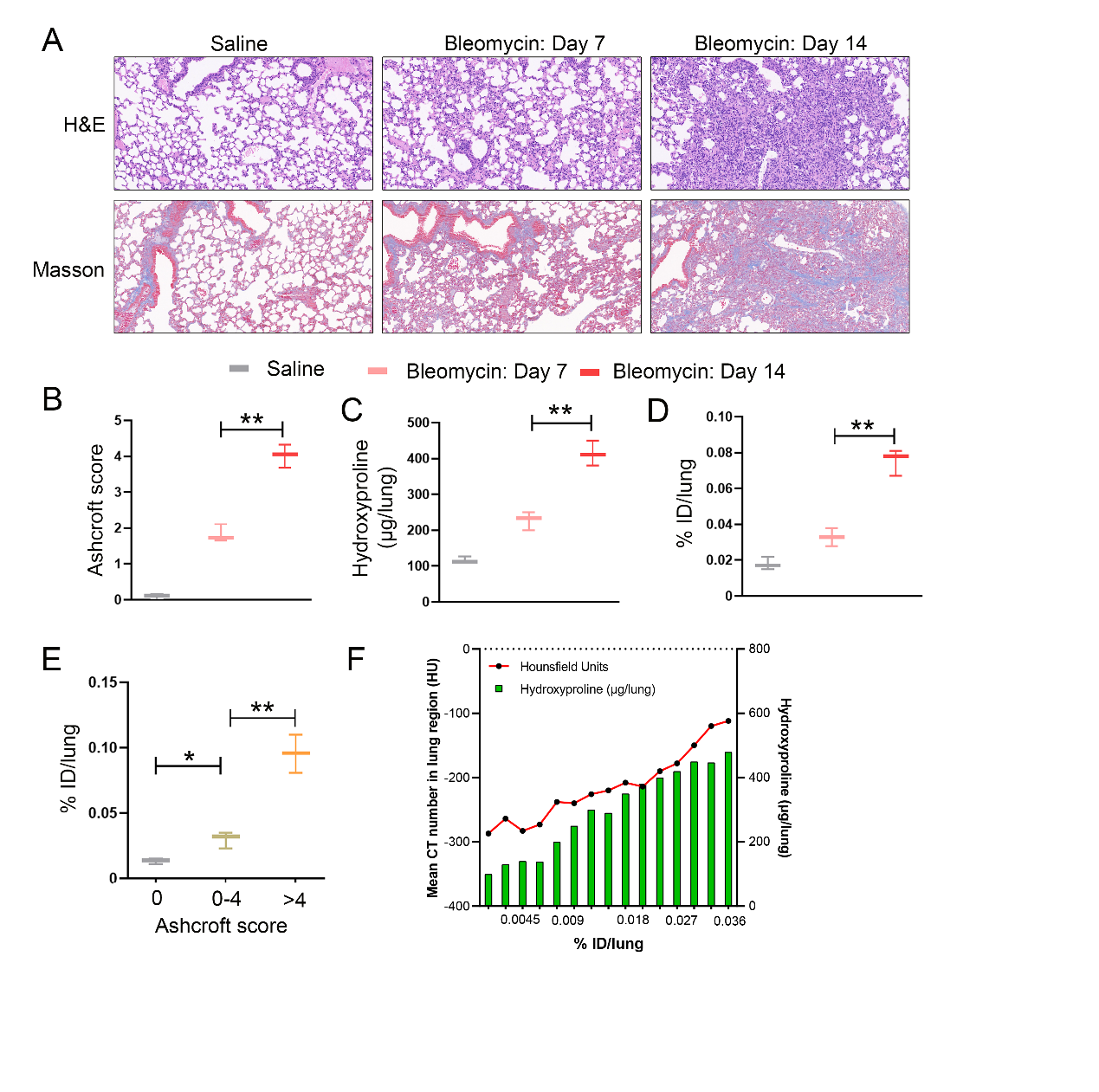


**Supplementary Figure S7. Iodine@NPs monitors disease progression in the bleomycin-induced mouse model of pulmonary fibrosis.** Mice (n = 5 in each group) received either saline or bleomycin intratracheally. Mice were sacrificed 7, 14, and 21 days later. (A) Pulmonary fibrosis was determined by hematoxylin-eosin (H&E) staining and collagen was revealed by Masson’s trichrome staining. Disease progresses in a stepwise fashion as determined by histological Ashcroft scoring of lung fibrosis (B), and by hydroxyproline analysis (C). (n = 5; mean ± SD; **P < 0.01). (D) The uptake of iodine in lungs from mice 150 min after injection expressed as % ID/lung (n = 5; mean ± SD; **P < 0.01). (E) Correlation of iodine lung uptake and Ashcroft score (n = 5; mean ± SD; *P < 0.05, **P < 0.01). (F) The trend chart of the mean CT number in the lung region and hydroxyproline content with changes in iodine uptake in the lung.


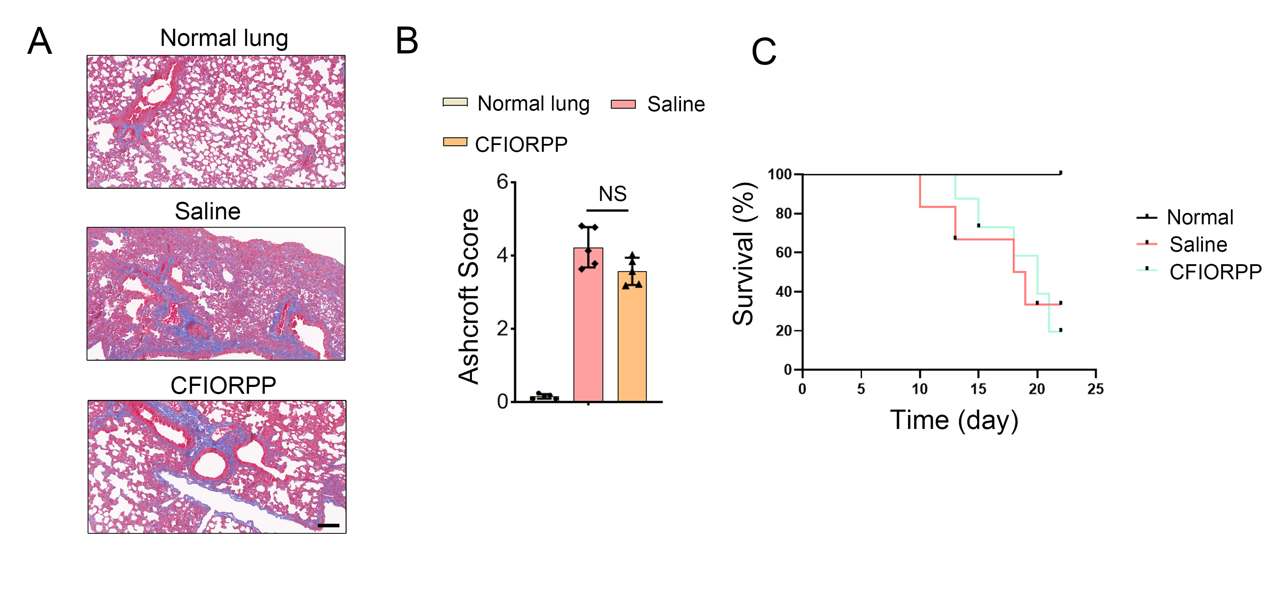


**Supplementary Figure S8. Administration of collagenase-modified (myo)fibroblast-targeting nanoparticles (NPs) in an advanced stage of pulmonary fibrosis does not hinder the progression of the disease.** Ten days after administration of bleomycin, mice were intravenously injected with saline or collagenase/Fab′@iodide@oltipraz/rosiglitazone@PEG-PAE nanoprobes (termed CFIORPP) every 3 days. Mice were sacrificed on the 21st day after bleomycin instillation. (A) Deposition of collagen I was visualized by Masson’s trichrome staining. Bar = 100 μm. (B) The quantification of pulmonary fibrosis is based on the Ashcroft score. Values are expressed as the mean ± SD (n = 5; *p < 0.05). NS, no significant. (C) Kaplan–Meier analysis for mice treated with saline, NC-NPs, (olti + rosi), or (olti + rosi)-NPs in the presence of bleomycin. Values are expressed as mean ± SD.


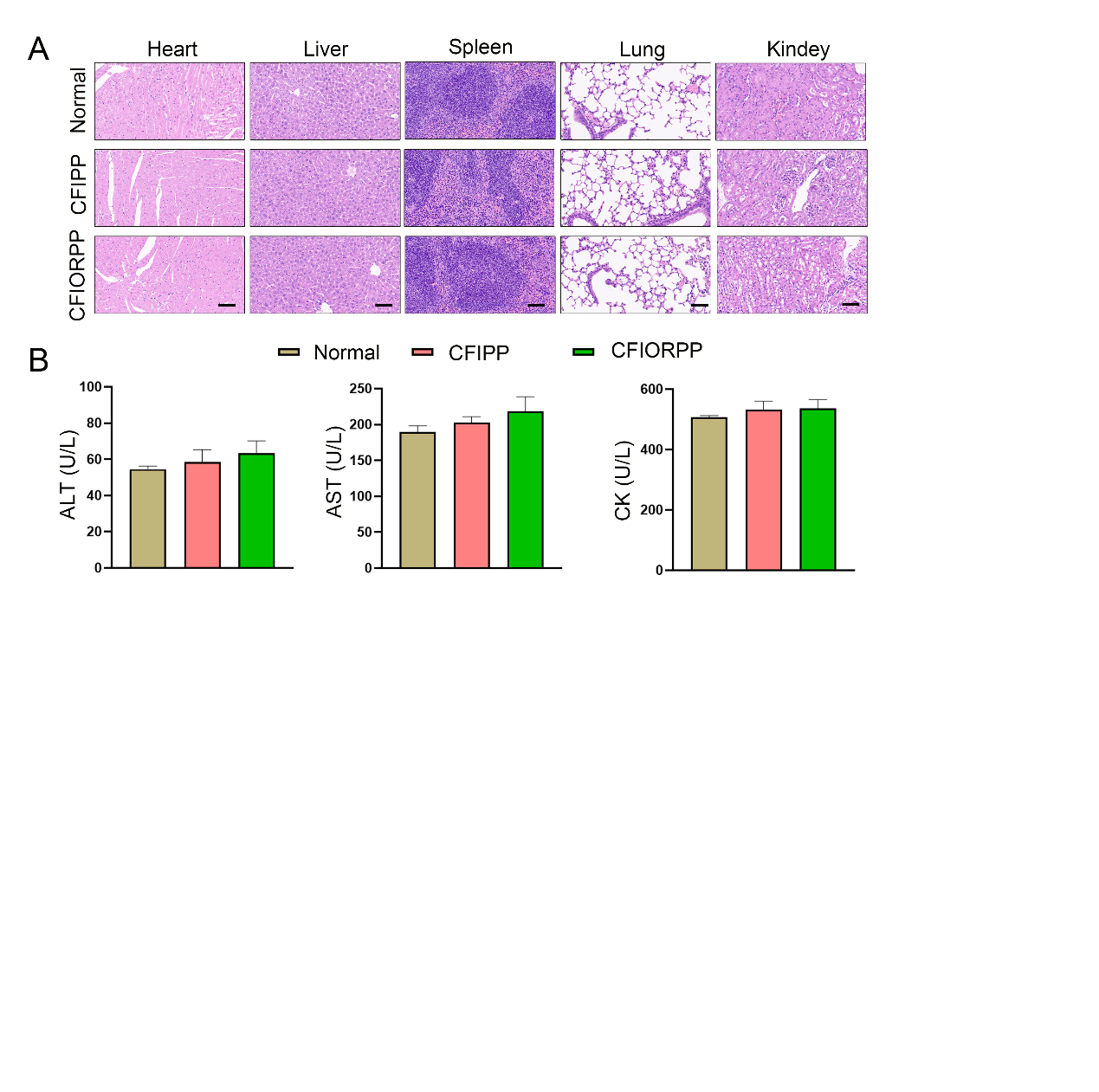


**Supplementary Figure S9. *In vivo* biosafety evaluation of the Collagenase/Fab’@iodide@oltipraz/rosiglitazone@PEG-PAE nanoprobe (termed CFIORPP).** (A) Histopathologic analyses of H&E-stained tissue sections from the major organs (heart, liver, spleen, lung, and kidney) of mice after the indicated treatment, Bar = 50 μm. (B) Biochemical analysis of mice treated with Saline (normal), CFIPP, or CFIORPP (ALT: alanine aminotransferase; AST: aspartate aminotransferase; CK: creatine phosphokinase). The data are presented as the means ± SD.
